# The role of optical coherence tomography in the evaluation of para-chiasmal lesions: a systematic review and meta-analysis

**DOI:** 10.3389/fopht.2025.1691582

**Published:** 2026-01-26

**Authors:** Khai Shin Alva Lim, Wen Xu Abel Tng, Wei De Bryan Theng, Bernett Teck Kwong Lee, Chee Fang Chin, Kelvin Zhenghao Li, Heather E. Moss

**Affiliations:** 1 Lee Kong Chian School of Medicine, Nanyang Technological University, Singapore, Singapore; 2 Yong Loo Lin School of Medicine, National University of Singapore, Singapore, Singapore; 3 Department of Ophthalmology, Tan Tock Seng Hospital, Singapore, Singapore; 4 Department of Ophthalmology, Stanford University, Palo Alto, CA, United States; 5 Department of Neurology and Neurosciences, Stanford University, Palo Alto, CA, United States

**Keywords:** pituitary, parachiasmal neoplasm, optical coherence tomography, retinal nerve fiber layer, ganglion cell layer, ganglion cell complex

## Abstract

**Introduction:**

While magnetic resonance imaging is currently the primary diagnostic tool for pituitary tumors, optical coherence tomography (OCT) may be used in evaluating the visual pathway impact of these lesions. This study evaluates the utility of OCT in patients with chiasmal compression from para-chiasmal lesions and determines its role in predicting visual field outcomes post-operatively.

**Methods:**

A search of five databases identified OCT studies in patients with neoplasms affecting the optic chiasm. Meta-analyses compared i) healthy controls versus patients, ii) good versus poor visual recovery post-operatively, and iii) patients with visual field defects (VFDs) versus those without. Standardized mean differences (SMDs) and mean differences (MDs) were used.

**Results:**

A review of 97 studies (5,300 patient eyes and 2,209 controls) demonstrated significantly thinner peripapillary retinal nerve fiber layer (pRNFL), macular RNFL (mRNFL), macular ganglion cell complex (mGCC), and macular ganglion cell–inner plexiform layer (mGCIPL) in patients as compared to controls. On pRNFL analysis, four-sector analysis demonstrated that patients had thinner RNFL in all quadrants compared to controls, with the greatest thinning in the inferior quadrant (MD −16.37 μm [−22.35, −10.39]) and the least in the nasal quadrant (MD −10.91 μm [−16.45, −5.38]). mRNFL analysis showed the greatest thinning in the supero-nasal (MD −11.57 μm [−19.32, −3.83]) and infero-nasal sectors (MD −11.39 μm [−17.38, −5.40]). The meta-analysis of mGCIPL sectors found the infero-nasal region to have the most thinning. Patients with good visual recovery had higher pre-operative mean pRNFL thickness (MD 11.35 μm [6.20, 16.49]).

**Discussion:**

Associations between OCT changes, neoplasms affecting the optic chiasm, and visual outcomes demonstrate its potential to support diagnosis and prognosis for patients with para-chiasmal lesions. Further research is needed to ascertain the relevance of pre-perimetric OCT changes.

## 1 Introduction

The optic chiasm is located superior to the pituitary gland and inferior to the hypothalamus (1). Due to their anatomical proximity, lesions of structures adjacent to the optic chiasm can result in visual field (VF) defects, the classical bitemporal hemianopia (2). In clinical practice, such VF defects can be assessed quantitatively using perimetry (3), while objective damage to the retinal ganglion cells can be assessed using non-invasive retinal imaging such as optical coherence tomography (OCT) (4). OCT utilizes infrared light to generate cross-sectional images of the eye at resolutions of 5–20 μm (5) and has been reported to be more sensitive for the detection of chiasmal impact by para-chiasmal lesions than visual field testing (6). Visual field testing and OCT complement magnetic resonance imaging (MRI) detect and characterize small soft tissue changes in the region of the chiasm (7, 8) by demonstrating the functional damage and microstructural visual pathway damage caused by para-chiasmal lesions, respectively. This study aimed to evaluate the utility of OCT in evaluating patients with chiasmal compression from para-chiasmal lesions compared to controls and to determine its role in predicting visual field outcomes post-operatively and monitoring patients pre-operatively.

## 2 Methods

### 2.1 Search strategy and information sources

The Preferred Reporting Items for Systematic Reviews and Meta-Analyses (PRISMA) reporting guidelines were utilized (9).

A search of PubMed, Embase, SCOPUS, CINAHL, and Web of Science was conducted from the inception of the databases until August 2024. An additional 24 papers from previous studies were also included in the search. The search terms and strategies can be found in the **Supplementary Material**. In addition, the reference lists of identified studies were reviewed, and any additional studies meeting the inclusion criteria were also included in the review.

### 2.2 Selection process and eligibility criteria

Two independent reviewers (KSAL and WXAT) assessed the studies for inclusion. The inclusion criteria were as follows: i) studies that utilized OCT; ii) studies with subjects with para-chiasmal neoplasms; iii) studies comparing subjects with and without para-chiasmal lesions (such as pituitary tumors, meningiomas, craniopharyngiomas, or Rathke’s cleft cysts) or subjects with good versus poor post-operative VF outcomes; and iv) studies that were published since 2010. The exclusion criteria were as follows: i) studies that were reviews, systematic reviews, meta-analyses, case reports, guidelines, letters, or protocols; ii) studies that were not in English; iii) studies that were not conducted in humans; and iv) studies where the number of eyes studied was less than 10. The number of eyes was selected based on studies suggesting that the minimum number of participants in a study should be nine (10) and to reduce the number of underpowered studies, which may introduce bias and heterogeneity in a meta-analysis (11).

### 2.3 Data extraction and analysis

Retrieved data were uploaded into EndNote X20 and imported into the COVIDENCE Systematic Review Software (Veritas Health Innovation, Melbourne, Australia) for screening. Inconsistencies during screening were resolved by discussion or by a third reviewer’s intervention.

Data extracted from the papers included

1. authors, year of publication, and sample size; and
2. patients’ characteristics and disease status.

OCT measurements included the peripapillary retinal nerve fiber layer (pRNFL), macular retinal nerve fiber layer (mRNFL), macular ganglion cell complex, and macular ganglion cell–inner plexiform layer (mGCIPL).

For each of two comparisons (patients vs. control, and good vs. poor visual field outcomes), meta-analyses were performed for OCT measurements reported in the form of mean and standard deviation in four or more studies. For other comparisons, OCT measurements reported in other formats (such as median or mean with an interquartile range) or OCT measurements reported in fewer than four studies, meta-analyses were not performed. If two studies report on the same study group but have differing outcomes, both studies may be included. However, if similar outcomes are reported, the studies may be excluded from analysis (12). If a study reported on the results of both eyes of a study subject, the better eye would be chosen to reduce the selection bias of significant results.

Meta-analyses for outcomes were conducted in RevMan, Version 5.4 (Nordic Cochrane Centre) to evaluate standardized mean differences (SMDs) and mean differences (MDs) for the parameters that were reported in mean and standard deviation. SMD allows for the comparison of parameters regardless of the OCT models or patient demographics, such as age and gender (13), while MD allows for the pooling of the average differences in OCT parameter thicknesses between the subjects studied (14). Results for SMD are reported in standard deviations (SDs), while MDs are reported in micrometers (μm).

Heterogeneity was assessed using *I*^2^, a statistic that describes the percentage of the variability in effect estimates due to heterogeneity rather than sampling errors, with low, moderate, and high levels set at 25%, 50%, and 75%, respectively (15). In cases of moderate or high levels of heterogeneity, a random-effects meta-analysis model was used; otherwise, a fixed-effects model was utilized.

Quality and risk of bias assessments were conducted using the QUADAS-2 tool, which assesses patient selection, index test, reference standard, flow, and timing of the diagnostic tests, along with applicability (16). Authors KSAL and WXAT independently assessed study bias. Disagreements were resolved with discussion or with a third-party review. The significance level of all tests was set at p < 0.05.

## 3 Results

### 3.1 Study selection

**Figure 1:**
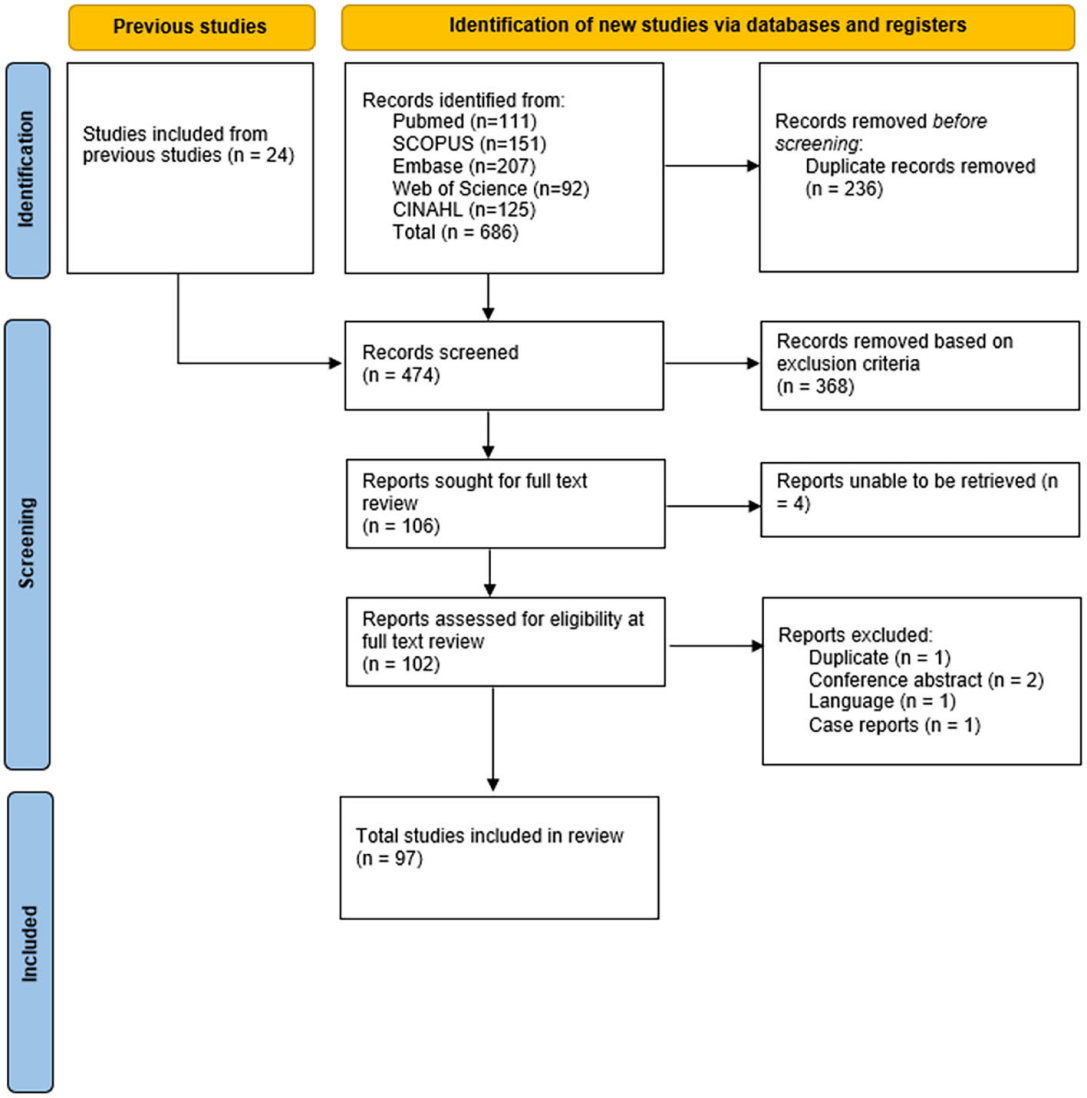
PRISMA study selection flowchart. PRISMA, Preferred Reporting Items for Systematic Reviews and Meta-Analyses.

A total of 710 studies were identified, 106 were sought for full-text review, and 97 were included for this review (**Figure 1**). Risk of bias information can be found in the **Supplementary Material**.

### 3.2 Study characteristics

**Figure 2:**
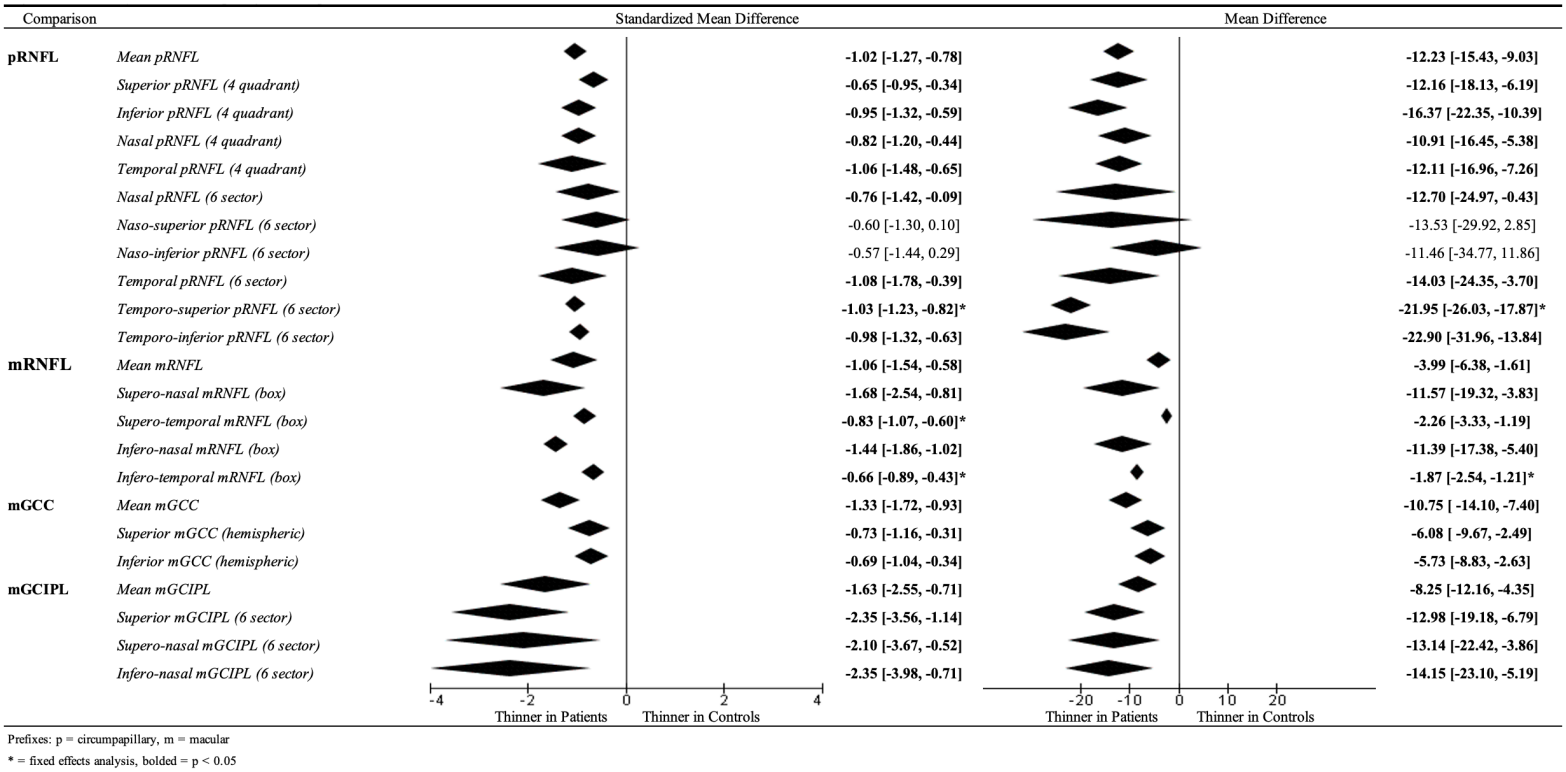
Meta-analysis results comparing patients and controls.

A total of 97 studies with a total of 5,300 eyes and 2,209 control eyes were reviewed (**Table 1**) (2, 17–112). Of these studies, 51 were not included in prior meta-analyses, and 43 studies had results that were utilized for the meta-analysis comparing patients and controls (**Figure 2**).

**Table 1:** Study characteristics.

Study authors	Comparison	Comparisons	Diagnosis	Control eyes	Patient eyes
Agarwal 2021 (17)	Case–control	Healthy vs. patient	PA	24	24
Akashi 2014 (19)	Case–control	Healthy vs. patient	Mixed	49	89
Akdogan 2022 (18)	Case–control	Healthy vs. patient, prolactin levels	PA	30	32
Altun 2017 (20)	Case–control	Healthy vs. patient, micro vs. macroadenoma	PA	72	68
Batur 2023 (21)	Case–control	Healthy vs. patient	PA	38	36
Bozzi 2024 (22)	Prospective	Over time	PA	40	
Cennamo 2015 (23)	Case–control	Healthy vs. patient	PA	43	33
Cennamo 2020 (112)	Case–control	Healthy vs. patient, pre- and post-op	PA	28	14
Cennamo 2021 (24)	Prospective	Over time, post-op	PA	16	
Chen 2023 (25)	Case–control	Healthy vs. patient	PA	45	24
Chou 2022 (26)	Case–control	Healthy vs. patient	PA	27	27
Chung 2020 (27)	Prospective	Pre- and post-op, normal and thin RNFL	PA	262	
Dallorto 2020 (28)	Case–control	Healthy vs. patient, optic neuropathy vs. no optic neuropathy	PA	17	16
Danesh Meyer 2015 (29)	Prospective	Over time, normal and thin RNFL	PA	213	
de Araujo 2017 (30)	Case–control	Healthy vs. patient	PA	30	37
Donaldson 2022 (2)	Retrospective	Healthy vs. patient, differing VFD, VFD vs. no VFD	Mixed	53	
Duru 2016 (31)	Case–control	Healthy vs. patient, micro vs. macroadenoma	PA	58	76
Ergen 2023 (32)	Case–control	Healthy vs. patient, chiasmal distortion	Mixed	210	92
Garcia 2014 (33)	Retrospective	Pre- and post-op	Mixed	38	
Ghezala 2021 (34)	Case–control and prospective	Healthy vs. patient, pre- and post-op, chiasmal compression	Mixed	24	32
Glebauskiene 2018 (35)	Case–control	Healthy vs. patient, suprasellar extension	PA	154	154
Hernandez-Echvarria 2022 (36)	Case–control and prospective	Healthy vs. patient, pre- and post-op	PA	57	35
Iegorova 2019 (37)	Case–control and retrospective	Healthy vs. patient, chiasmal compression	Mixed	76	20
Iqbal 2020 (38)	Prospective	Pre- and post-op	PA	40	
Jeon C 2019 (39)	Retrospective	Pre- and post-op, normal and thin RNFL	Mixed	216	
Jeon H 2021 (40)	Case–control	Healthy vs. patient, VFD vs. no VFD	Mixed	47	57
Jeon H 2022 (41)	Retrospective	Pre- and post-op, good and poor outcomes	Mixed	25	
Jorstad 2021 (42)	Prospective	Development of VFD, over time	Mixed	19	
Ju 2019 (43)	Retrospective	Optic tract edema vs. no edema	Mixed	212	
Kawaguchi 2019 (44)	Retrospective	Pre- and post-op, good and poor outcomes	Mixed	120	
Lei 2023 (46)	Case–control	Healthy vs. patient, compressive ON vs. glaucomatous ON	Mixed	72	36
Kurian 2022 (45)	Prospective	Pre- and post-op	PA	58	
Lang 2021 (47)	Case–control	Good and poor outcomes, controls used for rsfMRI	PA	42	19
Lee E 2015 (48)	Case–control	Healthy vs. patient, compressive ON vs. glaucomatous ON	Mixed	131	73
Lee G 2020 (1) (49)	Case–control	Healthy vs. patient, good and poor outcomes	Mixed	100	100
Lee G 2020 (2) (50)	Case–control	Healthy vs. patient, good and poor outcomes	Mixed	57	42
Lee G 2020 (3) (51)	Case–control	Healthy vs. patient	Mixed	36	35
Lee G 2021 (1) (52)	Case–control	Healthy vs. patient, pre- and post-op	Mixed	62	44
Lee J 2016 (53)	Retrospective	Pre- and post-op	CP	111	
Levchenko 2020 (54)	Case–control	Healthy vs. patient	PA	20	30
Li XC 2022 (55)	Case–control	PA vs. glaucoma	Mixed	74	
Loo 2013 (56)	Retrospective	Normal and thin RNFL	Mixed	14	
Mambour 2021 (57)	Retrospective	Pre- and post-op	Mixed	23	
Mangan 2021 (58)	Case–control	Healthy vs. patient, normal and thin RNFL, pre- and post-op	Mixed	29	14
Mavilio 2022 (59)	Case–control	Healthy vs. patient	PA	12	14
Mello 2022 (60)	Case–control	Healthy vs. patient	Mixed	33	40
Meyer 2022 (61)	Prospective	Good and poor outcomes	Mixed	216	
Mimouni 2019 (62)	Case–control	Compressive ON vs. glaucomatous ON	Mixed	31	
Monteiro 2010 (65)	Case–control	Healthy vs. patient	Mixed	35	35
Monteiro 2013 (63)	Case–control	Healthy vs. patient	PA	25	25
Monteiro 2014 (64)	Case–control	Healthy vs. patient	Mixed	33	36
Moon C-H (1) 2011 (66)	Prospective	Healthy vs. patient, pre- and post-op	Mixed	19	20
Moon C-H (2) 2011 (67)	Prospective	Healthy vs. patient, pre- and post-op	Mixed	18	20
Moon J 2020 (110)	Case–control	Healthy vs. patient	PA	47	22
Moura 2010 (68)	Case–control	Healthy vs. patient	Mixed	40	40
Nair 2024 (69)	Prospective	Pre- and post-op, good and poor outcomes	PA	66	
Nakamura 2012 (70)	Case–control	Healthy vs. patient	Mixed	26	64
Ogmen 2021 (71)	Case–control	Healthy vs. patient	PA	63	36
Ohkubo 2012 (72)	Case–control	Healthy vs. patient (post-op)	Mixed	23	33
Orman 2021 (111)	Case–control	Healthy vs. patient (pre-VFD)	PA	41	35
Ozcan 2022 (73)	Case–control	Healthy vs. patient, pre- and post-op	PA	17	17
Pang 2022 (75)	Case–control	Healthy vs. patient	PA	29	29
Pang 2023 (76)	Case–control	Healthy vs. patient, chiasmal compression	PA	106	106
Pang 2024 (77)	Retrospective	Pre- and post-op	PA	56	
Park 2021 (78)	Retrospective	Good and poor outcomes	PA	144	
Pekel 2014 (79)	Case–control	Healthy vs. patient	PA	60	60
Phal 2016 (74)	Prospective	Pre- and post-op, good and poor outcomes	Mixed	17	
Poczos 2019 (80)	Prospective	Pre- and post-op	Mixed	32	
Poczos 2022 (81)	Prospective	Pre- and post-op	Mixed	32	
Qiao 2016 (1) (82)	Prospective	Pre- and post-op, transsphenoidal vs. transcranial	PA	42	
Qiao 2016 (2) (83)	Prospective	Pre- and post-op	PA	46	
Rudman 2024 (84)	Retrospective	Pre- and post-op	PA	78	
Sahin 2017 (85)	Case–control	Healthy vs. patient	PA	33	40
Santorini 2021 (86)	Case–control	VFD vs. no VFD	Mixed	88	
Sasagawa 2023 (87)	Case–control	Chiasmal compression	Mixed	138	
Saxena 2015 (88)	Prospective	Pre- and post-op	PA	39	
Shinohara 2022 (89)	Retrospective	Pre- and post-op, optic nerve bending	Mixed	50	
Singha 2024 (90)	Retrospective	Pre- and post-op, VFD vs. no VFD	Mixed	70	
Sousa 2017 (91)	Case–control	Healthy vs. patients	PA	27	43
Suh 2021 (92)	Retrospective	VFD vs. no VFD	Mixed	41	
Sun 2017 (94)	Case–control	Healthy vs. patients	PA	38	39
Sun 2020 (93)	Case–control	Healthy vs. patients	PA	38	43
Suzuki 2020 (95)	Case–control	Healthy vs. patients	Mixed	33	42
Tang 2022 (1) (96)	Case–control	Good and poor outcomes	PA	25	25
Tang 2022 (2) (97)	Case–control	Healthy vs. patients, chiasmal compression	PA	100	41
Thammakumpee 2022 (98)	Retrospective	Pre- and post-op, VFD vs. no VFD, good vs. poor outcome	PA	36	
Tieger 2017 (99)	Case–control	Healthy vs. patients	Mixed	45	45
Ueda 2015 (100)	Case–control	Healthy vs. patients	Mixed	53	72
Wang G 2021 (101)	Case–control	Healthy vs. patients, VFD vs. no VFD	Mixed	31	34
Wang M 2020 (102)	Prospective	Pre- and post-op	Mixed	462	
Wang M 2021 (103)	Prospective	Pre- and post-op, normal and thin RNFL	Mixed	456	
Wang X 2022 (104)	Case–control	Healthy vs. patients	PA	40	40
Xia 2022 (105)	Case–control	Pre- and post-op, good and poor outcomes	PA	81	
Yang 2016 (106)	Case–control	Healthy vs. patients	Mixed	64	64
Yoneoka 2015 (107)	Prospective	Pre- and post-op, good and poor outcomes	Mixed	70	
Yoo 2020 (108)	Retrospective	Good and poor outcomes	Mixed	79	
Yum 2016 (109)	Retrospective	Healthy vs. patient vs. NTG	PA	77	32
Total				5,300	2,209

NTG, normal tension glaucoma; ON, Optic neuropathy; PA, Pituitary adenoma; RNFL, retinal nerve fiber layer; rsfMRI, resting state functional magnetic resonance imaging; VFD, visual field defect.

OCT devices that were used included Zeiss Cirrus OCT (26 studies), Zeiss Stratus OCT (nine studies), Topcon DRI OCT (seven studies), Topcon OCT (seven studies), Nidek RS-3000 (five studies), Optovue OCT (five studies), Optopol Revo (one study), Optovue RTVue (17 studies), Heidelberg Spectralis OCT (24 studies), and OTI Spectral OCT (one study) (**Table 2**). Among the 97 studies, 83 studies utilized spectral-domain OCT (SD-OCT), nine utilized time-domain OCT (TD-OCT), and seven utilized swept-source OCT. Seven studies utilized two devices in their analysis of patients (28, 44, 52, 70, 72, 84, 86). Due to the small number of studies utilizing time-domain and swept-source OCT, subgroup analysis was not performed. Two articles were unclear regarding the device studied (22, 69), while another article likely had a typo in the device name, and a search of the articles within the same department revealed that it utilized the Nidek RS-3000 (84).

**Table 2:** Summary of optical coherence tomography scan technology.

Study authors	Scan model	Technology utilized
Agarwal 2021 (17)	Zeiss Cirrus HD 4000	Spectral domain
Akashi 2014 (19)	Topcon OCT 2000	Spectral domain
Akdogan 2022 (18)	Optovue RTVue XR Avanti	Spectral domain
Altun 2017 (20)	Heidelberg Spectralis OCT	Spectral domain
Batur 2023 (21)	Heidelberg Spectralis OCT	Spectral domain
Bozzi 2024 (22)	Unclear	Unclear
Cennamo 2015 (23)	Optovue RTVue-100	Spectral domain
Cennamo 2020 (112)	Optovue	Spectral domain
Cennamo 2021 (24)	Optovue RTVue	Spectral domain
Chen 2023 (25)	Optovue RTVue XR Avanti	Spectral domain
Chou 2022 (26)	Optovue RTVue XR Avanti	Spectral domain
Chung 2020 (27)	Zeiss Cirrus HD	Spectral domain
Dallorto 2020 (28)	Zeiss Cirrus HD and Optovue RTVue XR Avanti	Spectral domain
Danesh Meyer 2015 (29)	Zeiss Stratus	Time domain
de Araujo 2017 (30)	Heidelberg Spectralis OCT	Spectral domain
Donaldson 2022 (2)	Zeiss Cirrus 600	Spectral domain
Duru 2016 (31)	Optovue RTVue-100	Spectral domain
Ergen 2023 (32)	Optovue RTVue XR Avanti	Spectral domain
Garcia 2014 (33)	Zeiss Stratus	Time domain
Ghezala 2021 (34)	Zeiss Cirrus HD 5000	Spectral domain
Glebauskiene 2018 (35)	Nidek RS-3000 Advance	Spectral domain
Hernandez-Echvarria 2022 (36)	Zeiss Cirrus 5000	Spectral domain
Iegorova 2019 (37)	Optopol Revo NX	Spectral domain
Iqbal 2020 (38)	Optovue	Spectral domain
Jeon C 2019 (39)	Heidelberg Spectralis OCT	Spectral domain
Jeon H 2021 (40)	Zeiss Cirrus HD	Spectral domain
Jeon H 2022 (41)	Zeiss Cirrus	Spectral domain
Jorstad 2021 (42)	Nidek RS-3000	Spectral domain
Ju 2019 (43)	Heidelberg Spectralis OCT	Spectral domain
Kawaguchi 2019 (44)	Nidek RS-3000 and Zeiss Stratus 3000	Spectral domain and time domain
Lei 2023 (46)	Optovue RTVue XR	Spectral domain
Kurian 2022 (45)	Topcon DRI OCT Triton plus	Swept source
Lang 2021 (47)	Zeiss Cirrus HD 4000	Spectral domain
Lee E 2015 (48)	Heidelberg Spectralis OCT	Spectral domain
Lee G 2020 (1) (49)	Heidelberg Spectralis OCT	Spectral domain
Lee G 2020 (2) (50)	Zeiss Cirrus HD	Spectral domain
Lee G 2020 (3) (51)	Zeiss Cirrus HD	Spectral domain
Lee G 2021 (1) (52)	Zeiss Cirrus HD OCT and Topcon DRI OCT Triton Plus	Spectral domain and swept source
Lee J 2016 (53)	Zeiss Cirrus HD	Spectral domain
Levchenko 2020 (54)	Zeiss Cirrus HD 5000	Spectral domain
Li XC 2022 (55)	Topcon DRI OCT Triton	Swept source
Loo 2013 (56)	Zeiss Stratus	Time domain
Mambour 2021 (57)	Zeiss Cirrus HD 5000	Spectral domain
Mangan 2021 (58)	OTI Spectral OCT	Spectral domain
Mavilio 2022 (59)	Zeiss Cirrus 500	Spectral domain
Mello 2022 (60)	Topcon DRI OCT Triton plus	Swept source
Meyer 2022 (61)	Heidelberg Spectralis OCT	Spectral domain
Mimouni 2019 (62)	Zeiss Stratus	Time domain
Monteiro 2010 (65)	Topcon OCT 1000	Spectral domain
Monteiro 2013 (63)	Topcon OCT 1000	Spectral domain
Monteiro 2014 (64)	Topcon OCT 1000	Spectral domain
Moon C-H (1) 2011 (66)	Zeiss Cirrus	Spectral domain
Moon C-H (2) 2011 (67)	Zeiss Cirrus	Spectral domain
Moon J 2020 (110)	Heidelberg Spectralis OCT	Spectral domain
Moura 2010 (68)	Zeiss Stratus	Time domain
Nair 2024 (69)	Unclear	Spectral domain
Nakamura 2012 (70)	Zeiss Cirrus and Optovue RTVue	Spectral domain
Ogmen 2021 (71)	Heidelberg Spectralis OCT	Spectral domain
Ohkubo 2012 (72)	Optovue RTVue-100 and Zeiss Stratus	Spectral domain and time domain
Orman 2021 (111)	Heidelberg Spectralis OCT	Spectral domain
Ozcan 2022 (73)	Nidek RS-3000 Advance	Spectral domain
Pang 2022 (75)	Heidelberg Spectralis OCT	Spectral domain
Pang 2023 (76)	Heidelberg Spectralis OCT	Spectral domain
Pang 2024 (77)	Heidelberg Spectralis OCT	Spectral domain
Park 2021 (78)	Zeiss Cirrus HD	Spectral domain
Pekel 2014 (79)	Heidelberg Spectralis OCT	Spectral domain
Phal 2016 (74)	Zeiss Stratus	Time domain
Poczos 2019 (80)	Heidelberg Spectralis OCT	Spectral domain
Poczos 2022 (81)	Heidelberg Spectralis OCT	Spectral domain
Qiao 2016 (1) (82)	Optovue RTVue	Spectral domain
Qiao 2016 (2) (83)	Optovue RTVue	Spectral domain
Rudman 2024 (84)	Zeiss Cirrus and Heidelberg Spectralis	Spectral domain
Sahin 2017 (85)	Heidelberg Spectralis OCT	Spectral domain
Santorini 2021 (86)	Optovue RTVue and Heidelberg Spectralis	Spectral domain
Sasagawa 2023 (87)	Nidek RS-3000	Spectral domain
Saxena 2015 (88)	Zeiss Stratus	Time domain
Shinohara 2022 (89)	Zeiss Cirrus	Spectral domain
Singha 2024 (90)	Heidelberg Spectralis OCT	Spectral domain
Sousa 2017 (91)	Topcon OCT 1000	Spectral domain
Suh 2021 (92)	Zeiss Cirrus	Spectral domain
Sun 2017 (94)	Topcon DRI OCT-1	Swept source
Sun 2020 (93)	Topcon DRI OCT-1 Atlantis	Swept source
Suzuki 2020 (95)	Topcon DRI OCT Triton plus	Swept source
Tang 2022 (1) (96)	Optovue	Spectral domain
Tang 2022 (2) (97)	Optovue	Spectral domain
Thammakumpee 2022 (98)	Zeiss Cirrus 4000	Spectral domain
Tieger 2017 (99)	Zeiss Cirrus 4000/5000	Spectral domain
Ueda 2015 (100)	Topcon OCT 2000	Spectral domain
Wang G 2021 (101)	Optovue	Spectral domain
Wang M 2020 (102)	Heidelberg Spectralis OCT	Spectral domain
Wang M 2021 (103)	Heidelberg Spectralis OCT	Spectral domain
Wang X 2022 (104)	Optovue RTVue XR Avanti	Spectral domain
Xia 2022 (105)	Topcon OCT 2000	Spectral domain
Yang 2016 (106)	Optovue RTVue-100	Spectral domain
Yoneoka 2015 (107)	Optovue RTVue-100	Spectral domain
Yoo 2020 (108)	Heidelberg Spectralis OCT	Spectral domain
Yum 2016 (109)	Zeiss Cirrus HD	Spectral domain

**Figure 3:**
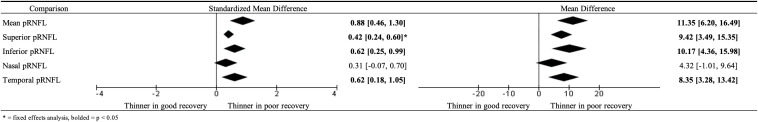
Meta-analysis results comparing visual function recovery vs. non-recovery.

In the 97 studies, a majority utilized the Humphrey Visual Field Perimeter; others utilized the Octopus Perimeter, Goldmann Perimeter, Kowa Perimeter, Centerfield Perimeter, MS Westfalia Perimeter, and the Vision Monitor Perimeter. Mean deviation was the most reported perimeter index and was used in 67 studies (**Table 3**). A total of 14 studies were utilized to compare good and poor visual field outcomes post-operatively (**Figure 3**). The studies that were not utilized for meta-analysis did not present the data in mean and standard deviation or had comparisons that did not have four or more studies.

**Table 3:** Summary of visual field assessment.

Study authors	Visual field assessment	Visual field assessment parameter
Agarwal 2021 (17)	Humphrey 30-2	Mean deviation
Akashi 2014 (19)	Humphrey 30-2	Mean deviation
Akdogan 2022 (18)	–	–
Altun 2017 (20)	–	–
Batur 2023 (21)	–	–
Bozzi 2024 (22)	Goldmann Kinetic Perimetry	Not reported
Cennamo 2015 (23)	Humphrey 30-2	Mean deviation, pattern standard deviation
Cennamo 2020 (112)	Humphrey 30-2	Mean deviation, pattern standard deviation
Cennamo 2021 (24)	–	–
Chen 2023 (25)	Octopus 101	Mean deviation
Chou 2022 (26)	Octopus 900	Mean deviation, mean sensitivity
Chung 2020 (27)	Humphrey 30-2	Mean deviation, visual field index
Dallorto 2020 (28)	Humphrey 24-2, Goldmann	Mean deviation
Danesh Meyer 2015 (29)	Humphrey 24-2	Mean deviation, pattern standard deviation
de Araujo 2017 (30)	Humphrey 24-2, 10-2	Mean deviation, test point sensitivity
Donaldson 2022 (2)	Humphrey 24-2	Mean deviation
Duru 2016 (31)	–	–
Ergen 2023 (32)	Humphrey 30-2	Mean deviation
Garcia 2014 (33)	Goldmann Kinetic Perimetry	Mean peripheral isopter area, mean peripheral isopter variation
Ghezala 2021 (34)	Humphrey 30-2	Mean deviation, pattern standard deviation
Glebauskiene 2018 (35)	Humphrey Full Field	Not reported
Hernandez-Echvarria 2022 (36)	Octopus 101 32	Mean deviation
Iegorova 2019 (37)	Centerfield 2 30-2	Mean deviation
Iqbal 2020 (38)	–	–
Jeon C 2019 (39)	Humphrey 30-2	Mean deviation
Jeon H 2021 (40)	Humphrey 30-2	Mean deviation
Jeon H 2022 (41)	Humphrey	Mean deviation
Jorstad 2021 (42)	Octopus 900 30	Mean deviation, square root of loss variance
Ju 2019 (43)	Humphrey 24-2	Mean deviation, visual field index
Kawaguchi 2019 (44)	Humphrey	Not reported
Lei 2023 (46)	Humphrey 24-2	Mean deviation
Kurian 2022 (45)	Humphrey 30-2	Mean deviation, visual field index
Lang 2021 (47)	Unclear 30-2	Mean deviation
Lee E 2015 (48)	Humphrey 24-2	Mean deviation
Lee G 2020 (1) (49)	Humphrey 30-2	Mean deviation
Lee G 2020 (2) (50)	Humphrey 30-2	Mean deviation
Lee G 2020 (3) (51)	Humphrey 30-2	Mean deviation
Lee G 2021 (1) (52)	Humphrey 30-2	Mean deviation
Lee J 2016 (53)	Humphrey 30-2	Mean deviation
Levchenko 2020 (54)	MS Westfalia 30-2	Mean deviation, pattern standard deviation
Li XC 2022 (55)	Octopus 900 G Standard	Mean deviation, square root of loss variance
Loo 2013 (56)	Humphrey	Mean deviation
Mambour 2021 (57)	Humphrey 24-2	Mean deviation, pattern standard deviation
Mangan 2021 (58)	Humphrey 24-2	Mean deviation
Mavilio 2022 (59)	Humphrey 24-2	Mean deviation, pattern standard deviation
Mello 2022 (60)	Humphrey 24-2	Mean deviation
Meyer 2022 (61)	Humphrey 24-2	Mean deviation
Mimouni 2019 (62)	–	–
Monteiro 2010 (65)	Humphrey 24-2	Mean deviation, visual field sensitivity loss
Monteiro 2013 (63)	Humphrey 24-2	Mean deviation
Monteiro 2014 (64)	Humphrey 24-2	Mean deviation, visual field sensitivity loss
Moon C-H (1) 2011 (66)	Humphrey 30-2	Mean deviation, pattern standard deviation, visual field sensitivity loss
Moon C-H (2) 2011 (67)	Humphrey 30-2	Mean deviation, pattern standard deviation, visual field sensitivity loss
Moon J 2020 (110)	Humphrey 24-2	Mean deviation
Moura 2010 (68)	Humphrey 24-2	Mean deviation, visual field sensitivity loss
Nair 2024 (69)	Goldmann Kinetic Perimetry	Subjective evaluation
Nakamura 2012 (70)	Humphrey 30-2	Mean deviation, temporal total deviation
Ogmen 2021 (71)	Unclear	–
Ohkubo 2012 (72)	Humphrey 24-2	Mean deviation, pattern standard deviation, visual field sensitivity loss
Orman 2021 (111)	Humphrey 30-2	Subjective evaluation
Ozcan 2022 (73)	Humphrey 24-2	Mean deviation, pattern standard deviation
Pang 2022 (75)	Unclear 30°	Mean deviation
Pang 2023 (76)	Kowa AP7000, 30°	Mean deviation
Pang 2024 (77)	Kowa AP7000, 30°	Mean deviation
Park 2021 (78)	Humphrey	Mean deviation, pattern standard deviation, visual field index
Pekel 2014 (79)	–	–
Phal 2016 (74)	Humphrey	Not reported
Poczos 2019 (80)	Humphrey 30-2	Mean deviation
Poczos 2022 (81)	Humphrey 30-2	Mean deviation
Qiao 2016 (1) (82)	Humphrey 30-2	Mean deviation
Qiao 2016 (2) (83)	Humphrey 30-2	Total deviation
Rudman 2024 (84)	Humphrey	Subjective evaluation
Sahin 2017 (85)	Humphrey 30-2	Mean deviation
Santorini 2021 (86)	Vision Monitor Kinetic Perimetry	Peripheral isopters
Sasagawa 2023 (87)	Humphrey 24-2	Mean deviation
Saxena 2015 (88)	Goldmann Kinetic Perimetry	Not reported
Shinohara 2022 (89)	–	–
Singha 2024 (90)	Unclear	Mean deviation
Sousa 2017 (91)	Humphrey 24-2	Individual test points
Suh 2021 (92)	Humphrey 30-2	Pattern standard deviation
Sun 2017 (94)	–	–
Sun 2020 (93)	–	–
Suzuki 2020 (95)	Humphrey 24-2, 10-2	Mean deviation, visual field sensitivity
Tang 2022 (1) (96)	Unclear	Mean deviation
Tang 2022 (2) (97)	Humphrey 24-2	Mean deviation
Thammakumpee 2022 (98)	Humphrey 24-2	Visual field index
Tieger 2017 (99)	Humphrey 30-2	Mean deviation
Ueda 2015 (100)	Humphrey 30-2	Mean deviation, visual field sensitivity
Wang G 2021 (101)	Octopus 900 central 30° and peripheral 30°–70°	Semiquantitative
Wang M 2020 (102)	Humphrey 24-2	Mean deviation, pattern standard deviation
Wang M 2021 (103)	Humphrey 24-2	Mean deviation, pattern standard deviation
Wang X 2022 (104)	Octopus 900	Mean deviation, mean sensitivity
Xia 2022 (105)	Humphrey 24-2	Mean deviation
Yang 2016 (106)	–	–
Yoneoka 2015 (107)	Goldmann Kinetic Perimetry	Isopters
Yoo 2020 (108)	Humphrey 24-2	Mean deviation, pattern standard deviation, visual field sensitivity
Yum 2016 (109)	Humphrey 24-2	Mean deviation, pattern standard deviation

### 3.3 OCT parameters in patients vs. controls

#### 3.3.1 Peripapillary retinal nerve fiber layer analysis

Retinal nerve fiber layer (RNFL) thickness scans were obtained at the optic nerve head in a circular linear scan for pRNFL analysis. Depending on the device used, the average thickness was split into four or six sectors. The four-sector scan was divided into superior, temporal, inferior, and nasal; the six-sector scan was divided into nasal, supero-nasal, supero-temporal, temporal, infero-temporal, and infero-nasal. A total of 38 papers compared the pRNFL thicknesses in patients versus controls (**Supplementary Figure 1, 29**). The mean pRNFL thickness was thinner in patients when compared to controls, with an SMD of −1.02 SD [−1.27, −0.78] (**Table 4**) and an MD of 12.23 μm [−15.43, −9.03].

**Table 4:** Results of the meta-analysis.

Comparison	Studies analyzing outcome (N)	standardized mean difference	P-value	Mean difference	P-value	Interpretation
Patients vs. healthy controls						
*pRNFL*						
Mean pRNFL	38	−1.02 [−1.27, −0.78]	<0.00001	−12.23 [−15.43, −9.03]	<0.00001	Thinner in patients
Superior pRNFL (4 quadrants)	19	−0.65 [−0.95, −0.34]	<0.0001	−12.16 [−18.13, −6.19]	<0.0001	Thinner in patients
Inferior pRNFL (4 quadrants)	17	−0.95 [−1.32, −0.59]	<0.00001	−16.37 [−22.35, −10.39]	<0.00001	Thinner in patients
Nasal pRNFL (4 quadrants)	18	−0.82 [−1.20, −0.44]	<0.0001	−10.91 [−16.45, −5.38]	0.0001	Thinner in patients
Temporal pRNFL (4 quadrants)	19	−1.06 [−1.48, −0.65]	<0.00001	−12.11 [−16.96, −7.26]	<0.00001	Thinner in patients
Nasal pRNFL (6 sectors)	4	−0.76 [−1.42, −0.09]	0.03	−12.70 [−24.97, −0.43]	0.04	Thinner in patients
Naso-superior pRNFL (6 sectors)	4	−0.60 [−1.30, 0.10]	0.09	−13.53 [−29.92, 2.85]	0.11	No significant difference
Naso-inferior pRNFL (6 sectors)	4	−0.57 [−1.44, 0.29]	0.19	−11.46 [−34.77, 11.86]	0.34	No significant difference
Temporal pRNFL (6 sectors)	4	−1.08 [−1.78, −0.39]	0.002	−14.03 [−24.35, −3.70]	0.008	Thinner in patients
Temporo-superior pRNFL (6 sectors)	4	−1.03 [−1.23, −0.82]*	<0.00001	−21.95 [−26.03, −17.87]*	<0.00001	Thinner in patients
Temporo-inferior pRNFL (6 sectors)	4	−0.98 [−1.32, −0.63]	<0.00001	−22.90 [−31.96, −13.84]	0.00001	Thinner in patients
*mRNFL*						
Mean mRNFL	5	−1.06 [−1.54, −0.58]	<0.0001	−3.99 [−6.38, −1.61]	0.001	Thinner in patients
Supero-nasal mRNFL (box)	4	−1.68 [−2.54, −0.81]	<0.0001	−11.57 [−19.32, −3.83]	0.003	Thinner in patients
Supero-temporal mRNFL (box)	4	−0.83 [−1.07, −0.60]*	<0.00001	−2.26 [−3.33, −1.19]	<0.0001	Thinner in patients
Infero-nasal mRNFL (box)	4	−1.44 [−1.86, −1.02]	<0.00001	−11.39 [−17.38, −5.40]	0.0002	Thinner in patients
Infero-temporal mRNFL (box)	4	−0.66 [−0.89, −0.43]*	<0.00001	−1.87 [−2.54, −1.21]*	<0.00001	Thinner in patients
*mGCC*						
Mean mGCC	19	−1.33 [−1.72, −0.93]	<0.00001	−10.75 [−14.10, −7.40]	<0.00001	Thinner in patients
Superior mGCC (hemispheric)	4	−0.73 [−1.16, −0.31]	0.0008	−6.08 [−9.67, −2.49]	0.0009	Thinner in patients
Inferior mGCC (hemispheric)	4	−0.69 [−1.04, −0.34]	0.0001	−5.73 [−8.83, −2.63]	0.0003	Thinner in patients
*mGCIPL*						
Mean mGCIPL	6	−1.63 [−2.55, −0.71]	0.0005	−8.25 [−12.16, −4.35]	<0.0001	Thinner in patients
Superior mGCIPL (6 sectors)	4	−2.35 [−3.56, −1.14]	0.0001	−12.98 [−19.18, −6.79]	<0.0001	Thinner in patients
Supero-nasal mGCIPL (6 sectors)	4	−2.10 [−3.67, −0.52]	p =0.009	−13.14 [−22.42, −3.86]	0.006	Thinner in patients
Infero-nasal mGCIPL (6 sectors)	4	−2.35 [−3.98, −0.71]	0.005	−14.15 [−23.10, −5.19]	0.002	Thinner in patients
Visual function recovery vs. non-recovery						
Mean pRNFL	8	0.88 [0.46, 1.30]	<0.0001	11.35 [6.20, 16.49]	<0.0001	Thicker in visual recovery
Superior pRNFL	7	0.42 [0.24, 0.60]*	<0.00001	9.42 [3.49, 15.35]	0.002	Thicker in visual recovery
Inferior pRNFL	7	0.62 [0.25, 0.99]	0.001	10.17 [4.36, 15.98]	0.0006	Thicker in visual recovery
Nasal pRNFL	6	0.31 [−0.07, 0.70]	0.11	4.32 [−1.01, 9.64]	0.11	No significant difference
Temporal pRNFL	7	0.62 [0.18, 1.05]	0.005	8.35 [3.28, 13.42]	0.001	Thicker in visual recovery

RNFL, retinal nerve fiber layer; GCC, ganglion cell complex; GCIPL, ganglion cell–inner plexiform layer.

Prefixes: p, circumpapillary, m, macular.

^*^Fixed-effects analysis.

In the four-sector pRNFL analysis, 20 studies were analyzed for the superior sectors, 18 studies for the inferior sectors, 19 studies for the nasal sectors, and 20 studies for the temporal sectors (**Supplementary Figures 2–5, 30–33**). In all of these studies, patients demonstrated thinner RNFL in every sector as compared to controls (**Table 4**). The inferior quadrant had the greatest thinning compared to the other sectors, with an MD of −16.37 μm [−22.35, −10.39]. The quadrant with the least thinning was the nasal quadrant with an MD of −10.91 μm [−16.45, −5.38].

In the six-sector pRNFL analysis, four studies were analyzed (**Supplementary Figures 6–11, 34–39**). When comparing between patients and healthy controls, the pRNFL in the nasal, temporal, supero-temporal, and infero-temporal sectors was significantly thinner in patients, as evidenced by the SMD and MD. In the nasal sector, the SMD was −0.76 SD [−1.42, −0.09], and the MD was −12.70 μm [−21.97, −0.43]. In the temporal sector, the SMD was −1.08 SD [−1.78, −0.39], and the MD was −14.03 μm [−24.35, −3.70]. The supero-temporal sector had an SMD of −1.03 SD [−1.23, −0.82] and an MD of −21.95 μm [−26.03, −17.87]. Lastly, the infero-temporal region had an SMD of −0.98 SD [−1.32, −0.63] and an MD of −22.90 μm [−31.96, −13.84] (**Table 4**). This difference in thinning was not observed in the supero-nasal and infero-nasal sectors (**Table 4**). In the supero-nasal sector, the SMD was −0.60 SD [−1.30, 0.10] and the MD was −13.53 μm [−29.92, 2.85]; in the infero-nasal sector, the SMD was −0.57 SD [−1.44, 0.29], and the MD was −11.46 μm [−34.77, 11.86].

#### 3.3.2 Macular retinal nerve fiber layer analysis

For mRNFL analysis, the scans were centered on the fovea. The mean thickness over the scanned area was analyzed in the form of a macular grid, after which data in the form of an Early Treatment of Diabetic Retinopathy Study (ETDRS) circle or as a box can be extracted. A total of 11 papers compared the macular RNFL between patients and controls (19, 30, 48, 49, 60, 64, 71, 93, 94, 110, 111). When analyzed using SMD and MD, the mean macular RNFL thicknesses were thinner in patients than in controls (**Supplementary Figure 12, 41**, **Table 4**).

In box analysis, all four sectors (supero-nasal, supero-temporal, infero-nasal, and infero-temporal) were thinner in patients (**Supplementary Figures 13–16, 41–44**). Four studies were included in this comparison. As compared to the other sectors, there was greater thinning of the mRNFL layer in patients in the supero-nasal sector with an MD of −11.57 μm [−19.32, −3.83] and the infero-nasal sector with an MD of −11.39 μm [−17.38, −5.40] as compared to healthy controls (**Table 4**).

There were insufficient papers for the analysis of subsectors presented in the ETDRS circle.

#### 3.3.3 Macular ganglion cell complex analysis

The macular ganglion cell complex (mGCC), which includes the three innermost retinal layers (i.e., the nerve fiber layer, the ganglion cell layer, and the inner plexiform layer) at the macula, was studied in 21 papers comparing patients to controls (2, 19, 23, 26, 28, 34, 36, 37, 46, 50, 51, 59, 60, 72, 94–96, 99, 104, 106, 112). These layers were analyzed by both the total mean values and the superior and inferior hemispheres of the mGCC (**Supplementary Figures 17–19, 45–47**). It was found that the mean thickness and hemispheric mGCC thickness were significantly thinner in patients as compared to controls on both MD and SMD analyses, with the superior mGCC being −6.08 μm [−9.67, −2.49] thinner and inferior mGCC being −5.73 μm [−8.83, −2.63] thinner (**Table 4**).

Four studies evaluated nasal and temporal hemispheric GCC; however, two studies reported on the same patient group with similar OCT results (60, 95); hence, this analysis could not be performed (12).

#### 3.3.4 Macular ganglion cell–inner plexiform layer analysis

There were 11 papers that studied the mGCIPL thickness measurement differences between patients and controls (17, 19, 40, 52, 54, 64, 73, 85, 93, 94, 109). Analysis was split into mean analysis and six circumferential sectoral analyses (**Supplementary 20–23, 48–52**). The mean mGCIPL was found to be thinner in patients as compared to healthy controls with an SMD of −1.63 SD [−2.55, −0.71] and an MD of −8.25 μm [−12.16, −4.35] (**Table 4**).

For sectoral analysis, meta-analysis was conducted for the superior, supero-nasal, and infero-nasal sectors, as these sectors met the analysis criteria requiring four or more studies with analyzable data. Meta-analysis revealed that patients had thinner superior, supero-nasal, and infero-nasal mGCIPL layers as compared to healthy controls (**Table 4**). The infero-nasal mGCIPL showed the greatest thinning with an MD of −14.15 μm [−23.10, −5.19], while the superior sector showed the least thinning with an MD of 12.98 μm [−19.18, −4.35].

#### 3.3.5 Macular ganglion cell layer analysis

There were 13 studies that evaluated ganglion cell layer thickness measurements, all of which were at the macula (20, 21, 30, 48, 49, 55, 75–77, 90, 108, 110, 111). However, for the comparisons studied by the 13 studies, none of the comparisons met our criteria requiring four or more papers presenting data amenable to meta-analysis. It was reported in seven studies that ganglion cell layer thicknesses were thinner in patients as compared to controls (20, 30, 48, 49, 75, 110, 111).

### 3.4 OCT parameters in good vs. poor VF outcomes

A total of 14 studies analyzed the differences in OCT measurements pre-operatively in patients who had good visual function recovery following operation versus those with poor or no recovery (33, 41, 44, 47, 49, 50, 57, 69, 78, 97, 98, 105, 107, 108). In eight of these 14 studies, the data provided by the studies allowed for meta-analysis, as they were presented in mean and standard deviation formats. pRNFL was analyzed using the mean pRNFL, as well as by the superior, temporal, nasal, and inferior sectors (**Supplementary Figures 24–28, 52–56**). Pre-operative mean pRNFL thicknesses were lower in patients who had poor VF recovery as compared to those with good recovery (**Table 4**). Patients with good visual recovery had a thicker RNFL than patients without good visual recovery, with an MD of 11.35 μm [6.20, 16.49]. On sectoral analysis, pRNFL measurements in the superior, inferior, and temporal quadrants were thicker in patients with good visual recovery as compared to patients with poor or no recovery, while the nasal pRNFL demonstrated a lack of difference between the two groups. For the superior quadrants, the SMD was 0.42 SD [0.24, 0.60] and the MD was 9.42 μm [3.49, 15.35]. In the inferior quadrants, the greatest difference was seen, with an SMD of 0.62 SD [0.25, 0.99] and an MD of 10.17 μm [4.35, 15.98]. The temporal quadrant had an SMD of 0.62 SD [0.18, 1.05] and an MD of 8.35 μm [3.28, 13.42].

### 3.5 Visual field defects versus no visual field defects

No studies met the criteria for the analysis comparing patients presenting with visual field defects against patients without visual field defects.

## 4 Discussion

We conducted a systematic review and meta-analysis of the existing literature to identify studies where OCT was utilized in para-chiasmal lesions, and we evaluated the utility of OCT in the diagnosis, prognostication, and monitoring of these patients. We also included the analysis of mRNFL sectorally, conducted a meta-analysis for pre-operative pRNFL in patients with good visual recovery, added more papers for the meta-analysis than other studies, and analyzed different device models and brands.

We found that OCT has a role in demonstrating the microstructural damage caused by the compression on the optic chiasm as seen by the reduction in thicknesses of the pRNFL, mRNFL, mGCC, and mGCIPL in patients as compared to controls. Furthermore, we observed that patients with better visual recovery had thicker pre-operative pRNFL, which may guide prognostication. Our findings further support existing literature (113) that there may be a role for OCT in the evaluation of patients with para-chiasmal lesions.

### 4.1 Update of meta-analysis to the existing literature

In our study, the results largely support a prior meta-analysis by Jeong in 2022 (113) and Chou in 2020 (114), who identified significant thinning in OCT parameters in patients with para-chiasmal lesions. We also sought to clarify the results obtained by the previous meta-analysis to ensure that the results are coherent. In updating the meta-analysis, we included 49 more papers for the mean pRNFL analysis. We split the analysis of pRNFL and mRNFL in contrast to Jeong, but we found that there was no significant difference between the use of either measurement. To our knowledge, our study is the first to meta-analyze the mRNFL sectorally, demonstrating sectoral thinning corresponding to that of the visual fields, potentially providing a better anatomical–functional measure corresponding to the damage caused by para-chiasmal lesions. We also updated the findings for mGCC and mGCIPL with 11 and three more papers added, respectively, as compared to Jeong’s paper, further substantiating the results of the analysis of mGCIPL by Jeong. We also further conducted analysis on sectoral measurements of the various OCT parameters to further identify pathological patterns seen in patients with para-chiasmal lesions. While there may be a role for OCT in the monitoring of patients prior to the development of visual field defects, this requires more evidence.

### 4.2 OCT’s role in the evaluation of patients

The role of OCT is to allow for a structural analysis of the retinal microstructure, which is not amenable to visualization through MRI or perimetry (115). Retinal thickness measurements may reflect axonal loss even possibly before visual field defects are present (116), potentially allowing for pre-perimetric monitoring of patients with radiologically diagnosed para-chiasmal lesions. The use of OCT has been suggested to be used in conjunction with an MRI in other conditions, such as multiple sclerosis, possibly as an alternative for monitoring the disease (117).

The advancement in the technology of OCT, in the form of SD-OCT and more recently swept-source OCT, allows for better segmentation of the nerve fiber layers for improved analysis as compared to TD-OCT (118). Our study found that a majority of authors utilized SD-OCT and that only a few studies utilized TD-OCT. Through the utilization of SMD, where the mean differences are transformed to a common scale, the differences between the OCT machines were accounted for in the analysis (14, 119), allowing for the generalization of the results (120). Furthermore, in a previous study by Colin et al., with manual adjustment in SD-OCT segmentation lines, the measurements are comparable to those of TD-OCT, allowing for comparison between trials utilizing different OCT machines (121).

#### 4.2.1 Patients versus controls

For patients with para-chiasmal lesions, our meta-analysis confirms that OCT parameters demonstrate significant thinning in patients when compared to controls. Through the use of the standardized mean difference, it is demonstrated that, regardless of the machine model used, patients have reduced OCT parameters compared to controls. On further analysis with mean differences, depending on the sector analyzed, an average of >10-μm thinning in pRNFL parameters, >5-μm thinning in mGCC, >8-μm thinning in GCIPL, and >1.87-μm thinning in mRNFL parameters were seen.

Sectorally, the nasal, naso-superior, and naso-inferior peripapillary fibers were demonstrated to have smaller magnitudes of thinning as compared to the temporal, temporo-superior, and temporo-inferior fibers in patients with para-chiasmal lesions as seen on SMD analysis. This corresponds to the classical bitemporal hemianopia caused by pituitary adenomas in view of the Garway–Heath map of the structural–functional relationship between the visual fields and the peripapillary nerve fiber layer (122–124).

This lower magnitude of thinning of the nasal pRNFL was also previously noted by the meta-analysis of Chou et al. (114). Previous understanding of how the nerve fiber layers enter the optic nerve head has been a topic of debate, with nerve fibers nasal to the optic disc entering the disc nasally, while those temporal to the optic disc but nasal to the macula do not have clear origins (125). Our findings are consistent with the Garway–Heath map. In the Garway–Heath map, the nasal pRNFL fibers correlated to a smaller portion of the nasal hemifield. Due to the large number of foveal fibers entering the optic nerve head temporally (126), this would likely account for the greater thinning in the temporal pRNFL as compared to the nasal pRNFL. Patients with bitemporal hemianopia would therefore have more thinning in the temporal optic nerve head fibers due to the nasal hemiretinal fibers entering the optic disc temporally.

Our updated meta-analysis contradicts the more recent analysis by Jeong et al., who noted that the nasal RNFL has greater magnitudes of thinning (113). In Jeong’s study, the analysis of the RNFL was conducted with both peripapillary RNFL and macular RNFL, which may have confounded the results. As the nasal pRNFL and nasal mRNFL do not correspond to the distribution of the nerve fibers (123), our outcomes differed from Jeong’s.

Furthermore, the analysis of the macular RNFL showed that when scanning the macula with a box-shaped configuration, the nasal sectors demonstrated greater thinning as compared to the temporal sectors. This corresponds to the crossing over of the nasal hemiretinal fibers at the optic chiasm (94). It is highlighted that the classical bitemporal hemianopia distribution of visual field defects seen on perimetry is in connection with the fovea; thus, this is congruent with our findings that nasal sectors at the macular RNFL are thinner than the temporal sectors. This may be easier to interpret than the peripapillary RNFL. However, since there were only four studies evaluating RNFL at the macula, more studies would be needed to support the role of nasal hemiretinal RNFL evaluation in patients with optic chiasm lesions, as well as retinotopic maps of the nerve fiber decussations.

### 4.3 OCT’s role in prognosis for VF recovery

Current prognostic factors, such as the patient’s age, pre-operative visual field deficits, visual acuity, and presence of optic disc atrophy, do not fully predict post-operative visual field recovery (33). As demonstrated in our study, OCT may be used to identify the potential for visual recovery following surgery. OCT parameters allow for the quantification of permanent axonal loss, which includes an additional measurement of damage made because of para-chiasmal lesions (78). As our study demonstrated, pre-operative pRNFL was thicker in patients with good visual field recovery as compared to those with poor visual recovery. Sectoral analysis suggests that thinning of the superior, inferior, and temporal sectors is seen in patients with poorer visual outcomes. This suggests that the permanent axonal loss may be less in patients with good visual field recovery, and sectoral analysis may be utilized to further prognosticate patients.

#### 4.3.1 Relationship between pre-operative visual deficits and VF recovery

In a prior study, Jeon et al. (41) found that the retinal thickness, including pRNFL and mGCIPL, did not show a relationship with post-surgical visual field defect (VFD) improvement. Jeon argued that in other studies, including a paper by Moon et al. (110), the pre-operative visual field and visual acuity were already significantly different between the two VF populations and thus were not representative of the OCT’s prognostic ability. However, this study demonstrates that most papers did not have patients with significant pre-operative differences in functional visual deficits between the visual recovery and non-recovery groups. In five out of eight included studies (41, 47, 78, 98, 105) that compared pre-operative visual acuity and mean differences in visual field, no significant pre-operative differences in these variables were observed. Two out of eight studies showed significant differences in pre-operative visual function (visual field and/or visual acuity) in patients with post-operative visual recovery and those without visual recovery (97, 108). Lastly, Garcia et al. did not compare pre-operative visual acuity and mean differences in visual fields (33).

In our study, we found that pre-operative mean pRNFL thickness was lower in patients with poor VF recovery as compared to those with good recovery. All sectors, other than the nasal sectors, were significantly thinner in patient groups that did not have visual recovery, win an MD ranging from 8.35 μm [3.28, 13.42] to 11.35 μm [6.20, 16.49] (**Table 4**). This suggests that pre-operative RNFL thickness may have a prognostic value.

#### 4.3.2 Identification of a cut-off for predicting visual field recovery

In our systematic review, several studies have attempted to identify cut-offs for predicting visual field recovery utilizing various parameters. For pRNFL and mRNFL, the authors identified various cut-offs (44, 45, 49, 57, 127). Lee’s study demonstrated the highest sensitivities of >80% for visual field recovery with cut-offs of 24.5, 17, 26, and 25.5 μm for the superior, temporal, nasal, and inferior sectors of mRNFL, respectively. Kawaguchi noted that the pRNFL thicknesses of the temporal quadrants being <49 μm had an odds ratio of 15.6 for poor visual outcome. However, there was no unified cut-off for pRNFL or mRNFL thicknesses that predicted visual recovery best. macular Ganglion Cell Layer (mGCL) was also analyzed by Lee, Yoo, and Moon (108, 110). The last author found sensitivities and specificities for visual field recovery of >100% with a cut-off of 30.6 μm for mGCL thickness. For mGCC, only Mambour et al. (57) looked at this parameter with an area under the curve of >0.9 for mGCC thickness of ≥67 μm and mRNFL thickness of ≥75 μm.

The wide range of cut-offs of the different parameters cited by the above authors presents challenges to the clinical application of a cut-off for the prognostication of likely poor post-operative outcomes. However, from the above studies, macular parameters appear to have the best potential for being good predictors of visual field recovery, as seen by the sensitivities and specificities being >80% reported by the studies (49, 108).

### 4.4 OCT’s role in monitoring disease progression

In the management of pituitary adenomas, visual impairments and related symptoms secondary to mass effect are an indication for surgery with goals to prevent the progression of symptoms and to reverse symptoms (128). Our systematic review identified the potential for OCTs to be utilized in monitoring patients as VFDs progress. In Orman’s study (111), it was found that prior to the development of VFDs on perimetry, macular OCT parameters were already shown to be thinner. This raises the possibility of using OCT as a pre-perimetric clinical monitoring tool to detect optic nerve damage when VF testing is unavailable, unreliable, or prior to true VF defects. Wang et al. (101) found that when comparing patients with sellar mass but without VFD against healthy controls, although the pRNFL of patients was thinner, it was not statistically significant. This was, however, suggested to be due to smaller sample sizes or due to varying degrees of disease progression.

### 4.5 Clinical utilization of OCT

Currently, in the monitoring and diagnosis of para-chiasmal lesions, MRI with contrast remains the gold standard tool (7). OCT has the benefits of convenience and safety as compared to the MRI, given the lack of contrast, ease of conduct, and price differentials. As this study has shown, patients with para-chiasmal lesions have lower retinal layer thicknesses as compared to controls. Baseline measurements could be obtained for these patients, and these patients could be followed up for changes over time, either in conservative management or post-operatively. This study supports the use of OCT in the work-up and monitoring of patients with para-chiasmal lesions, but more work needs to be conducted in multi-centered prospective studies to determine the sensitivity and specificity of the modality at various OCT parameter cut-offs, as well as further analysis of macular OCT parameters.

### 4.6 Limitations

Not all studies looked at the same parameters, resulting in fewer studies being available for meta-analysis. Furthermore, there appears to be considerable heterogeneity in the measurements obtained through OCT. The various studies included in this paper also utilized varying machines, which have different calibrations; hence, pooling through standardized mean differences was performed, which may overestimate the absolute differences (14).

Diagnostic odds ratios could not be calculated due to the lack of studies investigating sensitivity and specificity. Hence, more studies should be performed with emphasis on sensitivity and specificity.

As different models may have different scanning speeds, technologies for segmentation, and calibrations, pooling of the measurements was conducted instead, potentially limiting generalizability.

## 5 Conclusion

This updated systematic review and meta-analysis of OCT provides a balanced perspective, and our analysis identifies OCT as a potentially viable tool in the evaluation, prognostication, and possibly monitoring of lesions affecting the optic chiasm.

## Data Availability

Publicly available datasets were analyzed in this study. This data can be found here: PubMed, Embase, SCOPUS, CINAHL, and Web of Science.
