# Supplementary material for: The role of optical coherence tomography in the evaluation of para-chiasmal lesions: a systematic review and meta-analysis
Source: Front Ophthalmol (Lausanne). 2026 Jan 26;5:1691582. doi: 10.3389/fopht.2025.1691582 (PMC12883424; doi:10.3389/fopht.2025.1691582)
Supplement: Supplementary file 2 [file DataSheet2.docx]

Supplementary Material

**1. Supplementary Tables**

| Database | Search terms used | Results |
| --- | --- | --- |
| Pubmed | Pituitary Gland OR Pituitary Stalk OR Hypophysis OR Hypophysis Cerebri OR Pituitary OR Sellar OR suprasellar  AND  Meningioma OR Craniopharyngioma OR Glioma OR Adenoma OR macroadenoma OR adenocarcinoma OR tumour OR neoplasm OR “Cushings Disease”  AND  “OCT angiography” OR OCTA OR OCT-A OR OCT(A) OR “Optical coherence tomography angiography” OR “Optical coherence tomography”  Filters: human, english | 111  Retrieved on 25/8/24 |
| Embase | (Pituitary Gland OR Pituitary Stalk OR Hypophysis OR Hypophysis Cerebri OR Pituitary OR Sellar).mp.  AND  (Meningioma OR Craniopharyngioma OR Glioma OR adenoma OR adenocarcinoma OR tumour or neoplasm OR cushing disease).mp.  AND  (OCT angiography OR OCTA OR OCT-A OR Optical coherence tomography angiography OR Optical coherence tomography).mp. | 207  Retrieved 25/8/24 |
| SCOPUS | ( TITLE-ABS-KEY ( oct AND angiography OR octa OR oct-a OR optical AND coherence AND tomography AND angiography OR optical AND coherence AND tomography ) )  AND  ( TITLE-ABS-KEY ( ( pituitary AND gland ) OR ( pituitary AND stalk ) OR ( hypophysis ) OR ( hypophysis AND cerebri ) OR ( pituitary ) OR ( sellar ) ) )  AND  ( ( TITLE-ABS-KEY ( ( "Pituitary Gland" ) OR ( "Pituitary Stalk" ) OR ( "Hypophysis" ) OR ( "Hypophysis Cerebri" ) OR ( "pituitary" ) OR ( "sellar" ) ) ) ) | 151  Retrieved on 25/8/24 |
| Web Of Science | ((ALL=((("Pituitary Gland") OR ("Pituitary Stalk") OR ("Hypophysis") OR ("Hypophysis Cerebri") OR ("pituitary") OR ("sellar")) AND (("meningioma") OR ("craniopharyngioma") OR ("adenoma") OR ("macroadenoma") OR ("adenocarcinoma") OR (glioma) OR (“cushing disease”))))  AND  ALL=("OCT angiography" OR "OCTA" OR "OCT-A" OR "OCT(A)" OR "optical coherence tomography angiography" OR "optical coherence tomography")) | 92  Retrieved on 25/8/24 |
| CINAHL | TX ( ("Pituitary gland" OR "pituitary stalk" OR "hypophysis" OR "hypophysis cerebri" OR "pituitary" )  AND  TX ( meningioma OR craniopharyngioma OR glioma OR sellar OR "pituitary gland adenoma" OR "pituitary gland macroadenoma" OR "pituitary gland adenocarcinoma" OR “cushings disease”)  AND  TX ( "OCT angiography" OR OCTA OR OCT-A OR OCT(A) OR "optical coherence tomography angiography" OR "optical coherence tomography" ) | 125  Retrieved 25/8/24 |
| Total |  | 684 |

Supplementary Table 1: Search strategy

**2. Supplementary Figures**


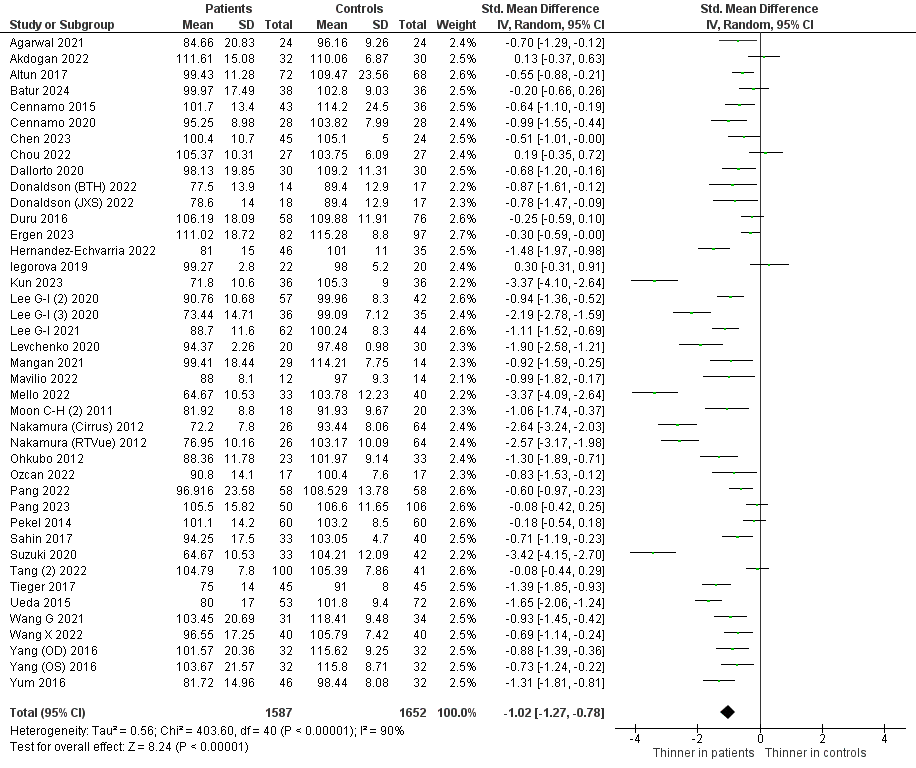


Supplementary figure 1 - SMD - healthy vs patients – mean pRNFL – Forest plot


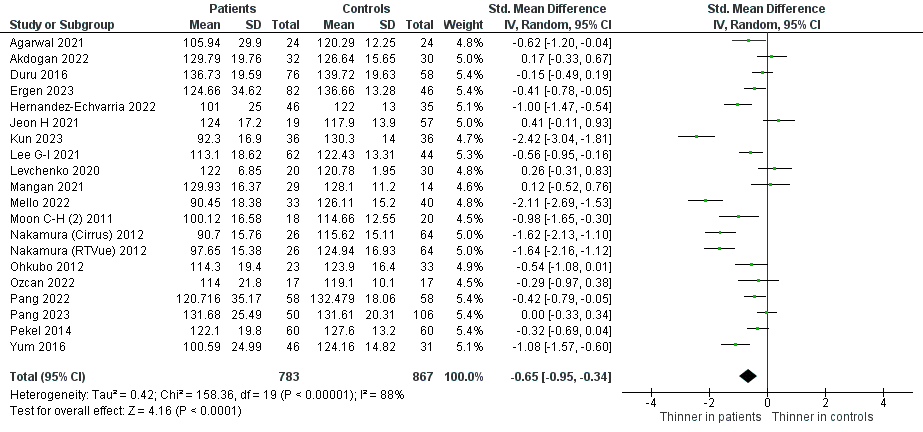


Supplementary figure 2 - SMD - healthy vs patients – superior pRNFL – Forest plot


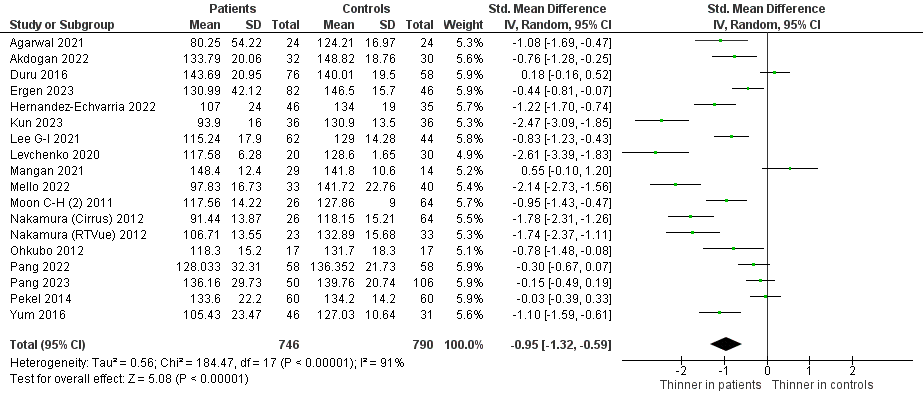


Supplementary figure 3 - SMD - healthy vs patients – inferior pRNFL – Forest plot


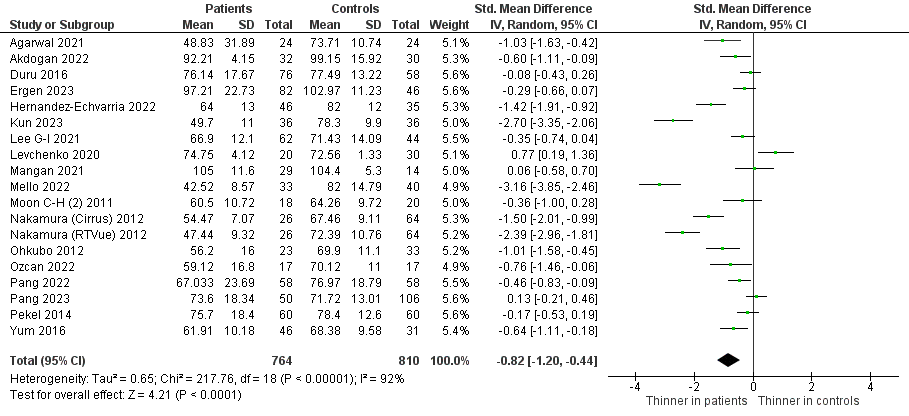


Supplementary figure 4 - SMD - healthy vs patients – nasal pRNFL – Forest plot


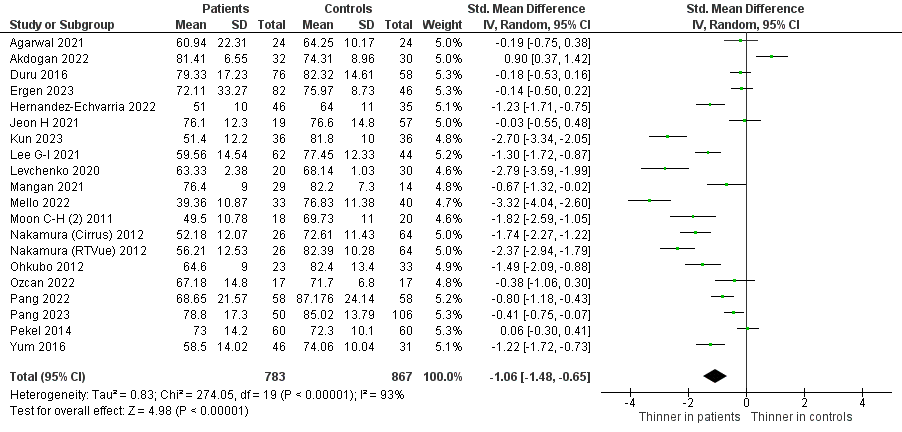


Supplementary figure 5 - SMD - healthy vs patients – temporal pRNFL – Forest plot


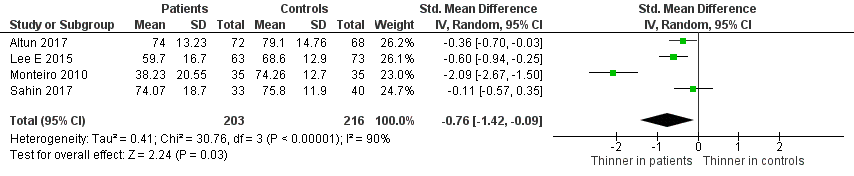


Supplementary figure 6 - SMD - healthy vs patients – nasal pRNFL (6 sector) – Forest plot


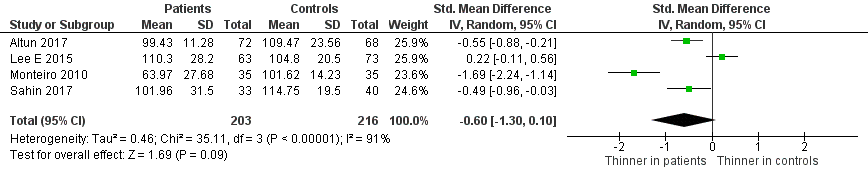


Supplementary figure 7 - SMD - healthy vs patients – naso-superior pRNFL (6 sector) – Forest plot


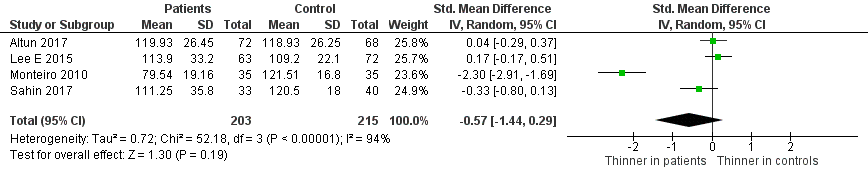


Supplementary figure 8 - SMD - healthy vs patients – naso-inferior pRNFL (6 sector) – Forest plot


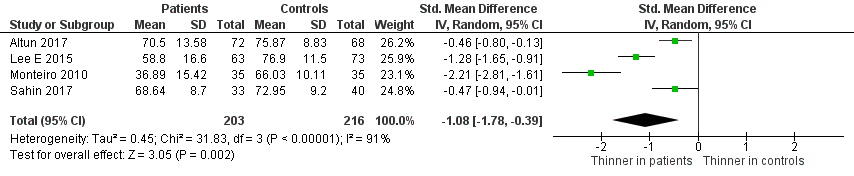


Supplementary figure 9 - SMD - healthy vs patients – temporal pRNFL (6 sector) – Forest plot


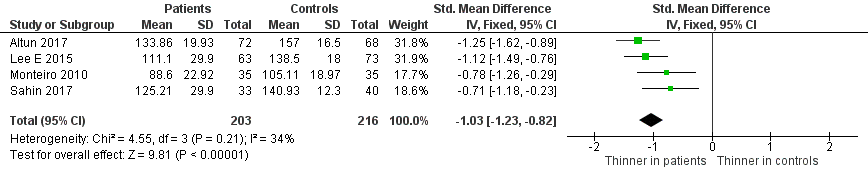


Supplementary figure 10 - SMD - healthy vs patients – temporo-superior pRNFL (6 sector) – Forest plot


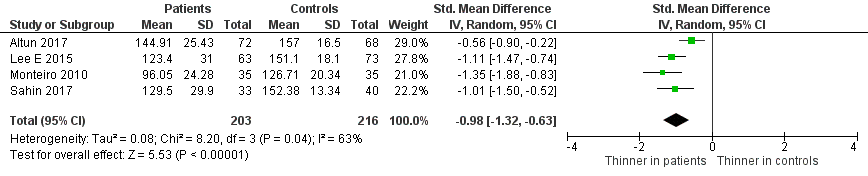


Supplementary figure 11 - SMD - healthy vs patients – temporo-inferior pRNFL (6 sector) – Forest plot


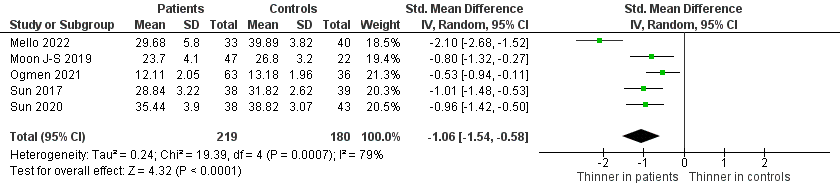


Supplementary figure 12 - SMD - healthy vs patients – mean mRNFL – Forest plot


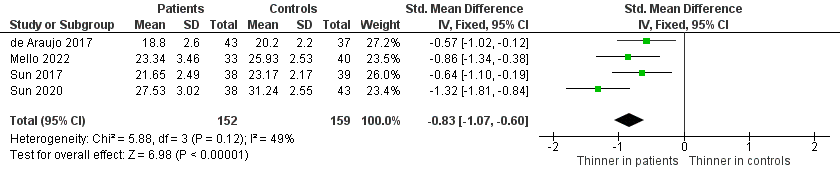


Supplementary figure 13 - SMD - healthy vs patients – supero-temporal mRNFL (box) – Forest plot


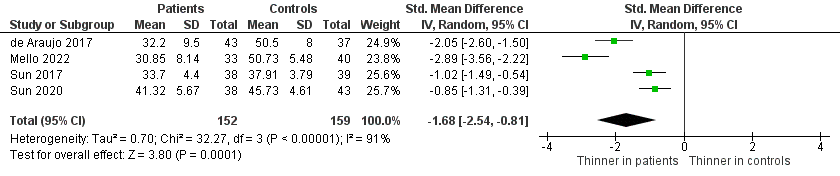


Supplementary figure 14 - SMD - healthy vs patients – supero-nasal mRNFL (box) – Forest plot


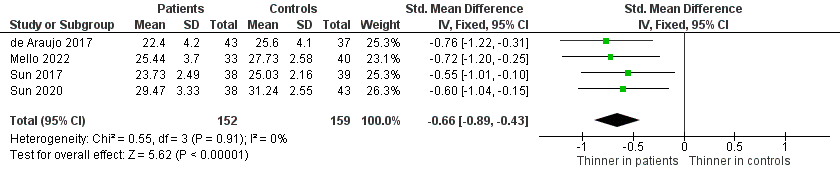


Supplementary figure 15 - SMD - healthy vs patients – infero-temporal mRNFL (box) – Forest plot


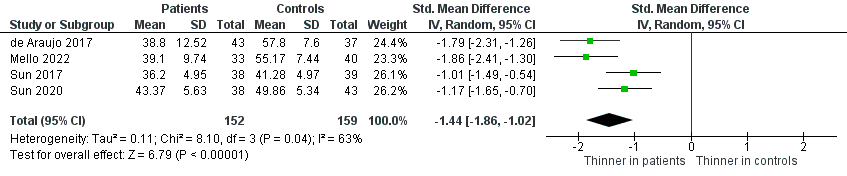


Supplementary figure 16 - SMD - healthy vs patients – infero-nasal mRNFL (box) – Forest plot


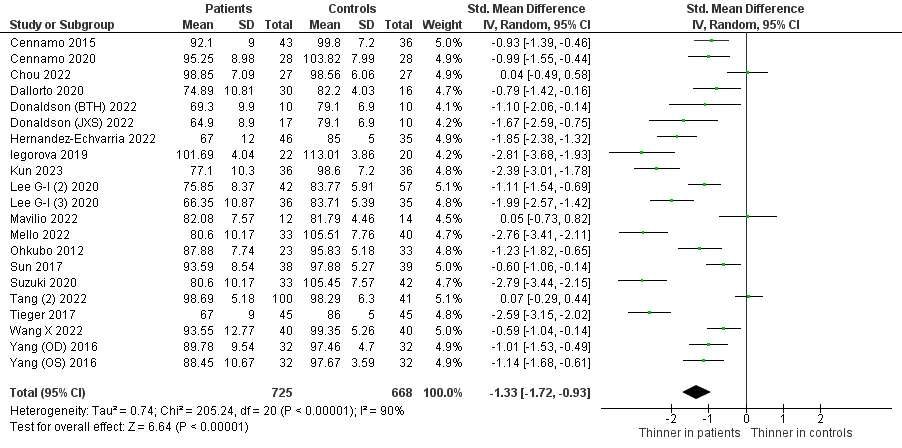


Supplementary figure 17 - SMD - healthy vs patients – mean mGCC – Forest plot


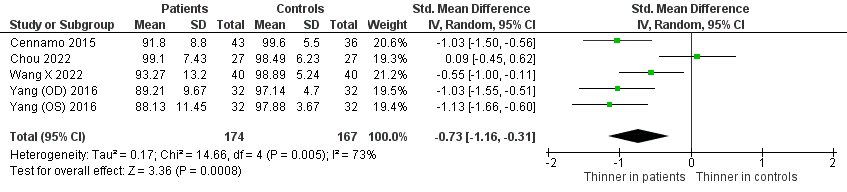


Supplementary figure 18 - SMD - healthy vs patients – superior mGCC – Forest plot


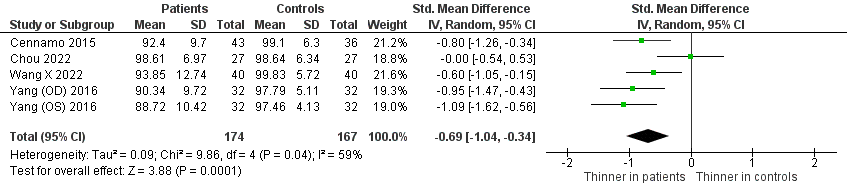


Supplementary figure 19 - SMD - healthy vs patients – inferior mGCC – Forest plot


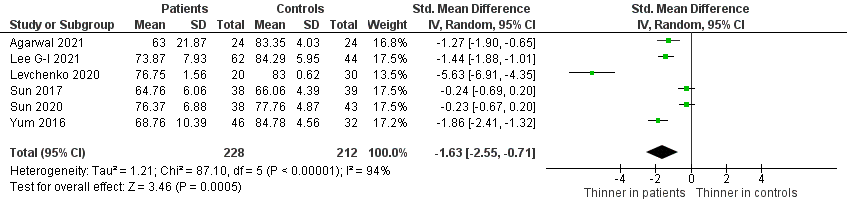


Supplementary figure 20 - SMD - healthy vs patients – mean mGCIPL – Forest plot


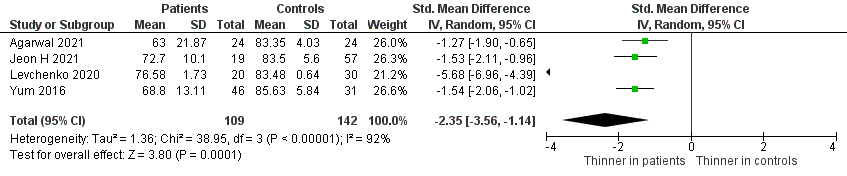


Supplementary figure 21 - SMD - healthy vs patients – superior mGCIPL (6 sector) – Forest plot


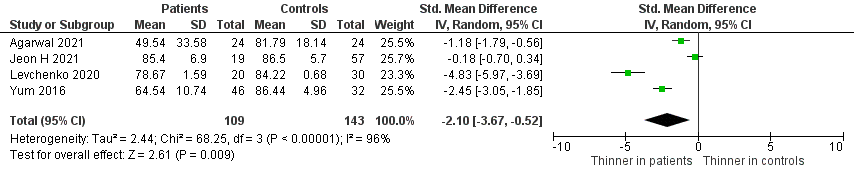


Supplementary figure 22 - SMD - healthy vs patients – supero-nasal mGCIPL (6 sector) – Forest plot


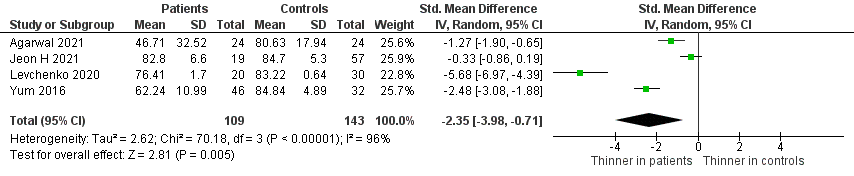


Supplementary figure 23 - SMD - healthy vs patients – infero-nasal mGCIPL (6 sector) – Forest plot


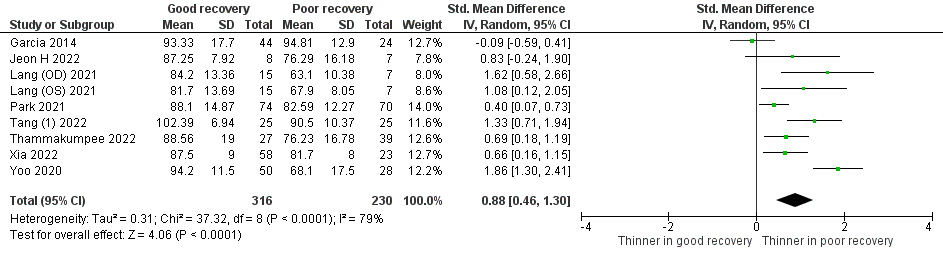


Supplementary figure 24 - SMD - good outcome vs poor outcome – mean pRNFL – Forest plot


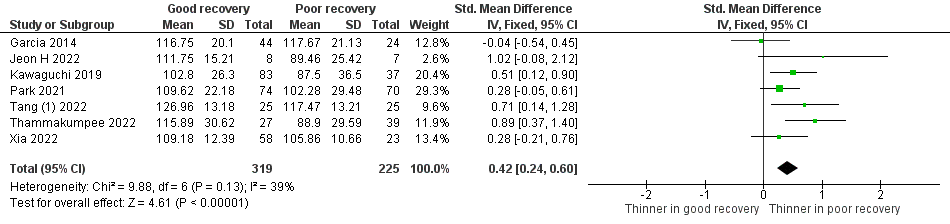


Supplementary figure 25 - SMD - good outcome vs poor outcome – superior pRNFL – Forest plot


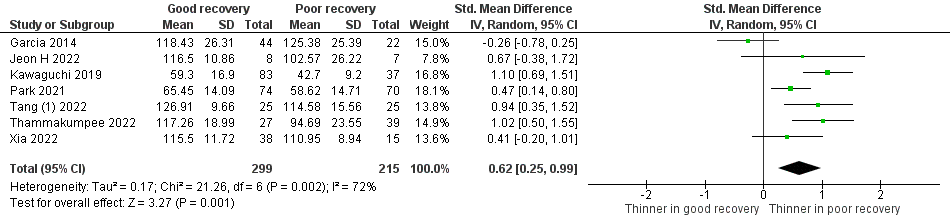


Supplementary figure 26 - SMD - good outcome vs poor outcome – inferior pRNFL – Forest plot


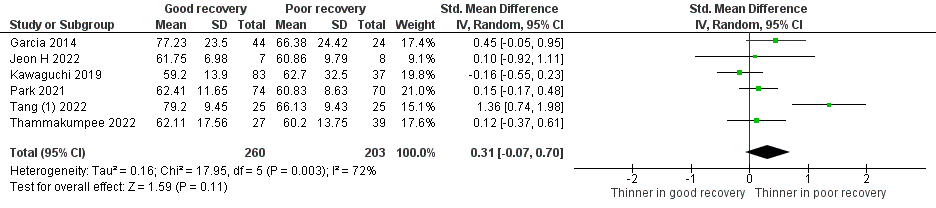


Supplementary figure 27 - SMD - good outcome vs poor outcome – nasal pRNFL – Forest plot


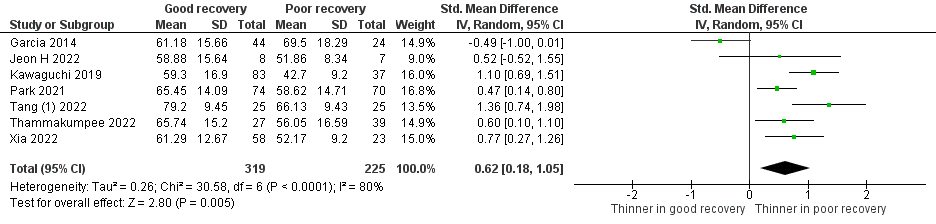


Supplementary figure 28 - SMD - good outcome vs poor outcome – temporal pRNFL – Forest plot


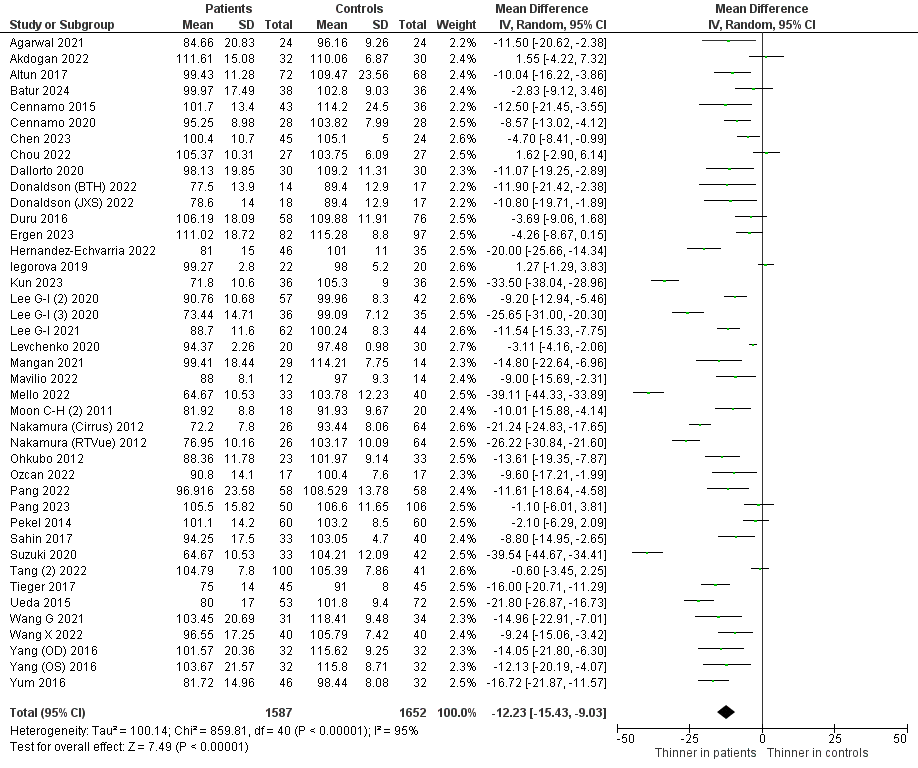


Supplementary figure 29 - MD - healthy vs patients – mean pRNFL – Forest plot


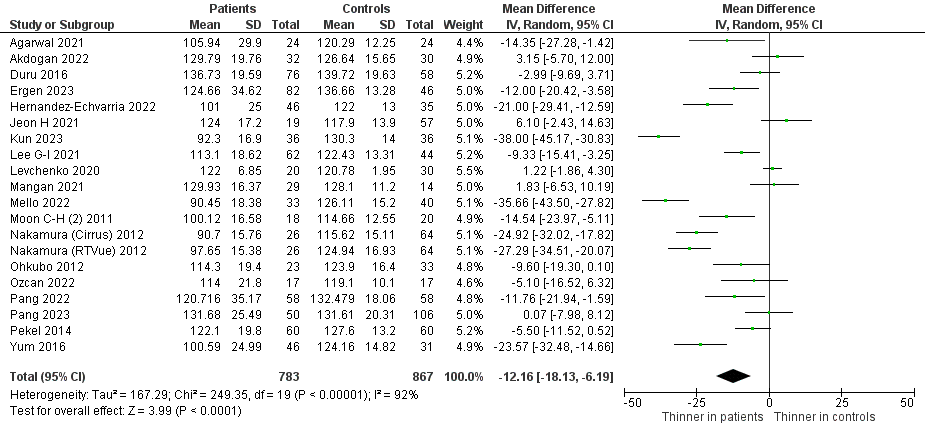


Supplementary figure 30 - MD - healthy vs patients – superior pRNFL – Forest plot


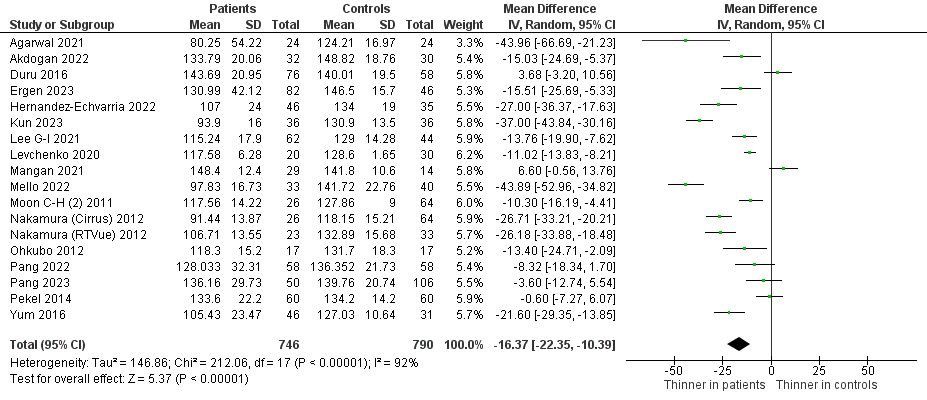


Supplementary figure 31 - MD - healthy vs patients – inferior pRNFL – Forest plot


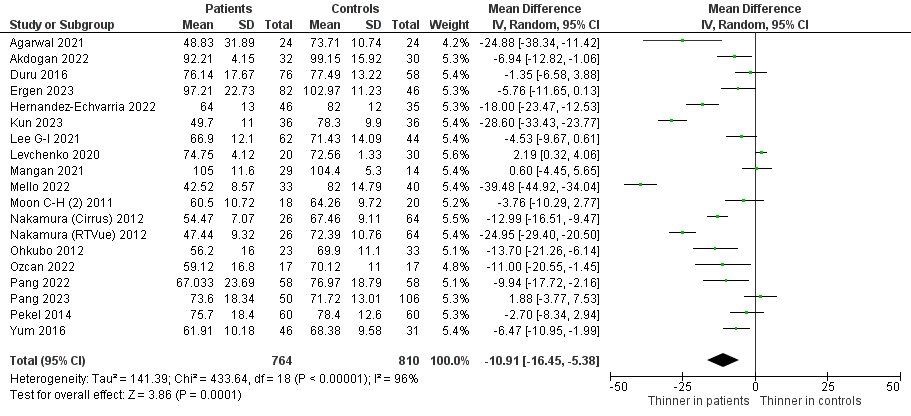


Supplementary figure 32 - MD - healthy vs patients – nasal pRNFL – Forest plot


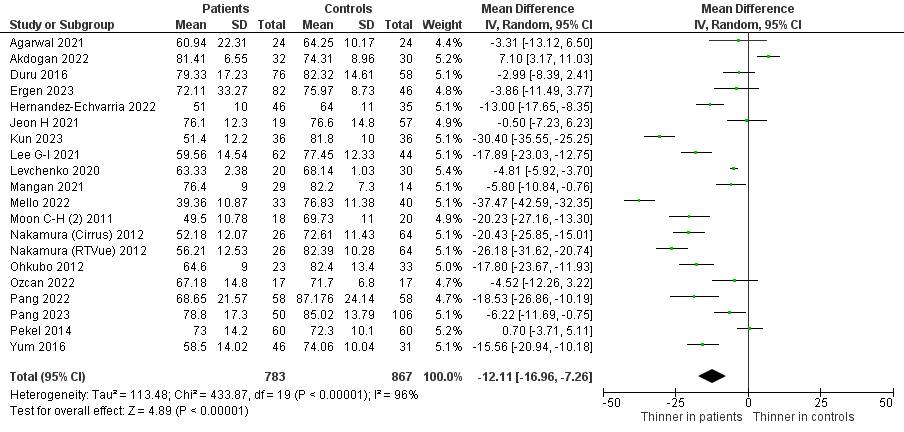


Supplementary figure 33 - MD - healthy vs patients – temporal pRNFL – Forest plot


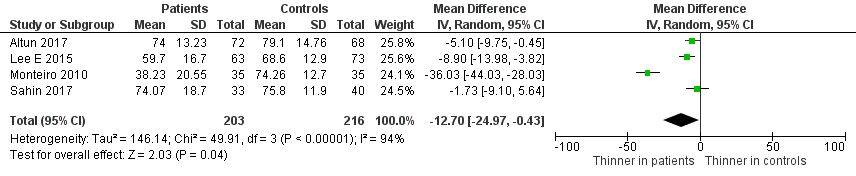


Supplementary figure 34 - MD - healthy vs patients – nasal pRNFL (6 sector) – Forest plot


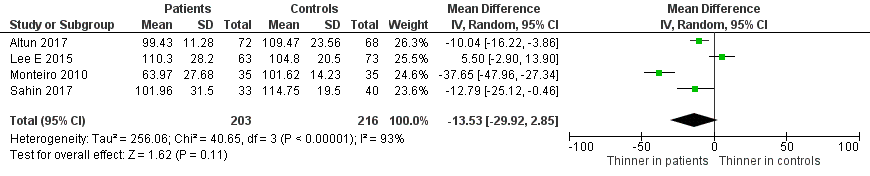


Supplementary figure 35 - MD - healthy vs patients – naso-superior pRNFL (6 sector) – Forest plot


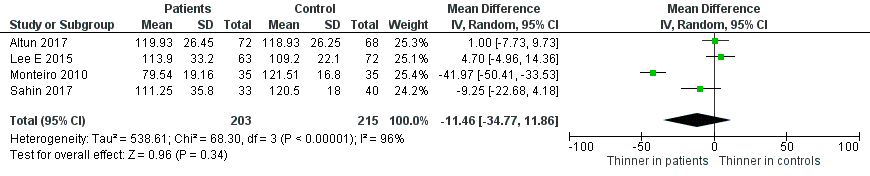


Supplementary figure 36 - MD - healthy vs patients – naso-inferior pRNFL (6 sector) – Forest plot


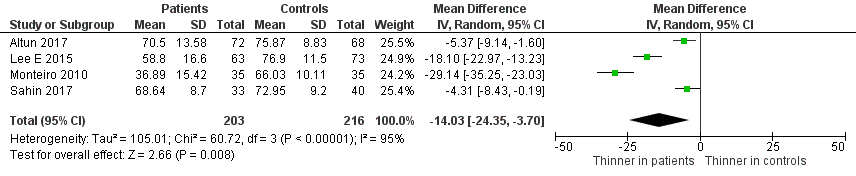


Supplementary figure 37 - MD - healthy vs patients – temporal pRNFL (6 sector) – Forest plot


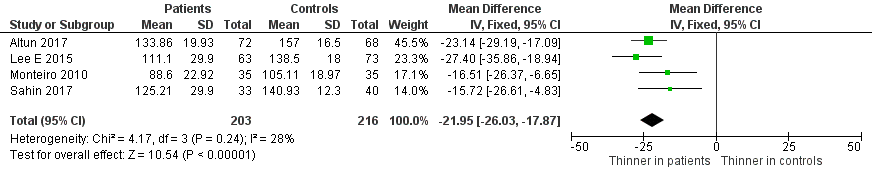


Supplementary figure 38 - MD - healthy vs patients – temporo-superior pRNFL (6 sector) – Forest plot


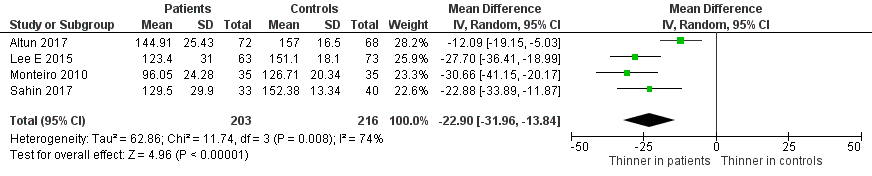


Supplementary figure 39 - MD - healthy vs patients – temporo-inferior pRNFL (6 sector) – Forest plot


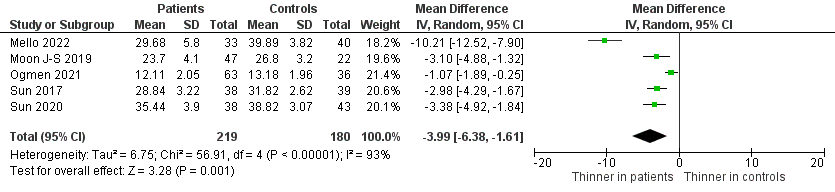


Supplementary figure 40 - MD - healthy vs patients – mean mRNFL – Forest plot


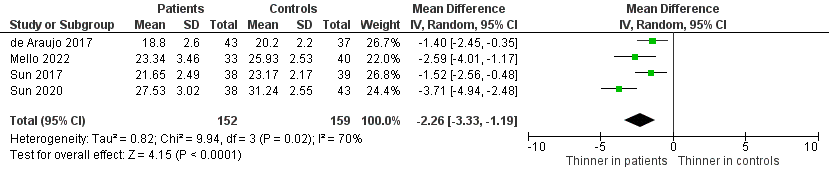


Supplementary figure 41 - MD - healthy vs patients – supero-temporal mRNFL (box) – Forest plot


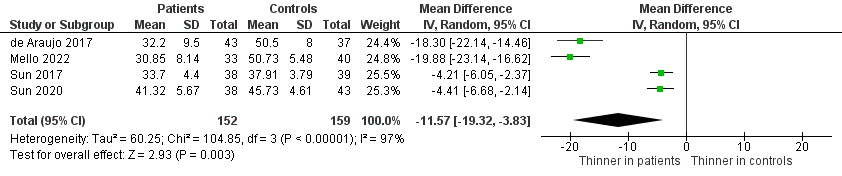


Supplementary figure 42 - MD - healthy vs patients – supero-nasal mRNFL (box) – Forest plot


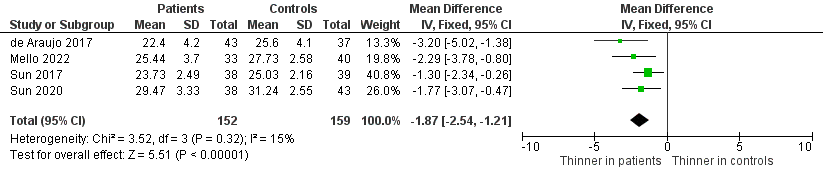


Supplementary figure 43 - MD - healthy vs patients – infero-temporal mRNFL (box) – Forest plot


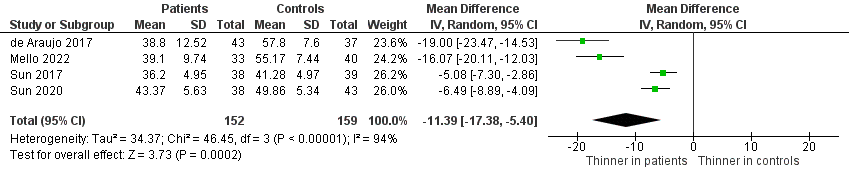


Supplementary figure 44 - MD - healthy vs patients – infero-nasal mRNFL (box) – Forest plot


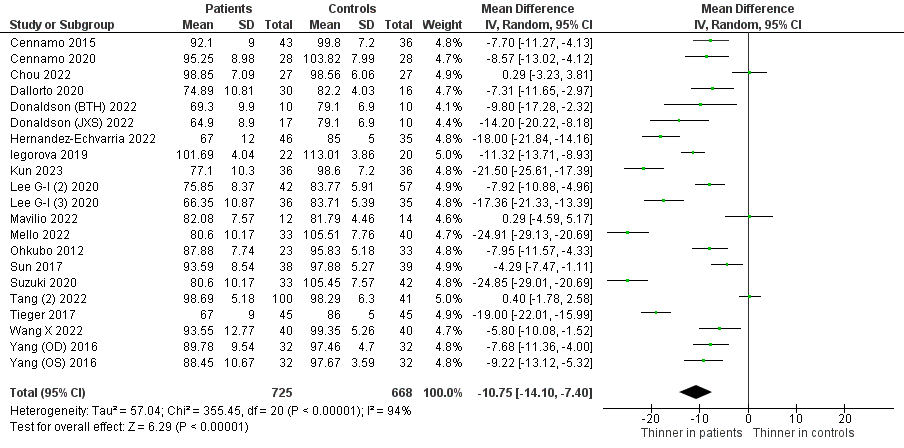


Supplementary figure 45 - MD - healthy vs patients – mean mGCC – Forest plot


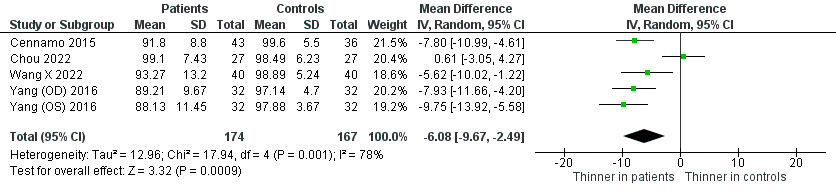


Supplementary figure 46 - MD - healthy vs patients – superior mGCC – Forest plot


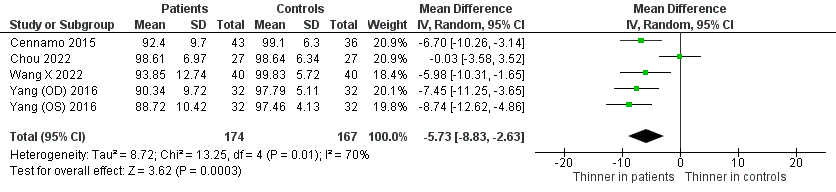


Supplementary figure 47 - MD - healthy vs patients – inferior mGCC – Forest plot


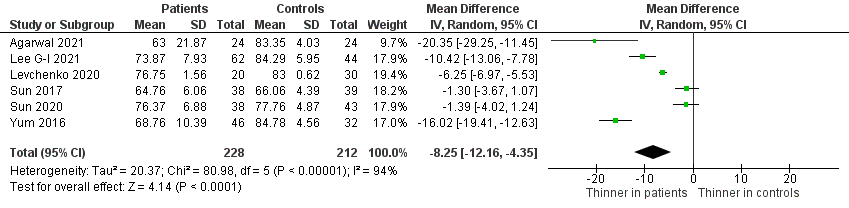


Supplementary figure 48 - MD - healthy vs patients – mean mGCIPL – Forest plot


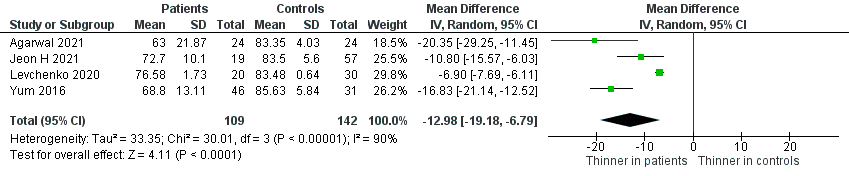


Supplementary figure 49 - MD - healthy vs patients – superior mGCIPL (6 sector) – Forest plot


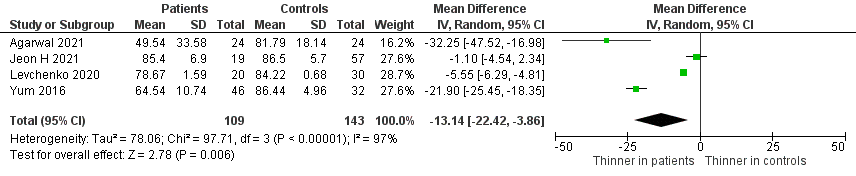


Supplementary figure 50 - MD - healthy vs patients – supero-nasal mGCIPL (6 sector) – Forest plot


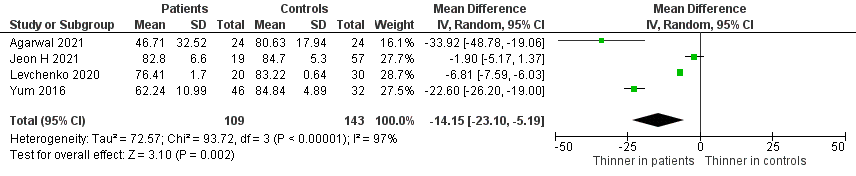


Supplementary figure 51 - MD - healthy vs patients – infero-nasal mGCIPL (6 sector) – Forest plot


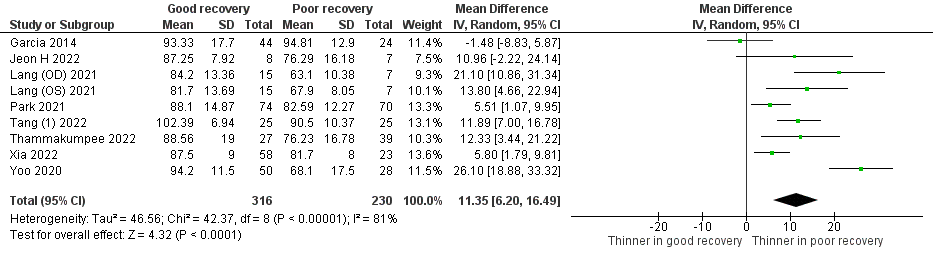


Supplementary figure 52 - MD - good outcome vs poor outcome – mean pRNFL – Forest plot


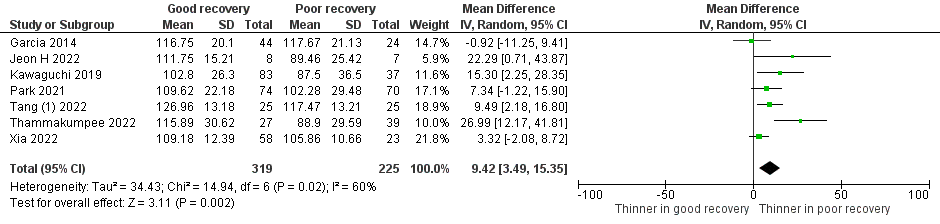


Supplementary figure 53 - MD - good outcome vs poor outcome – superior pRNFL – Forest plot


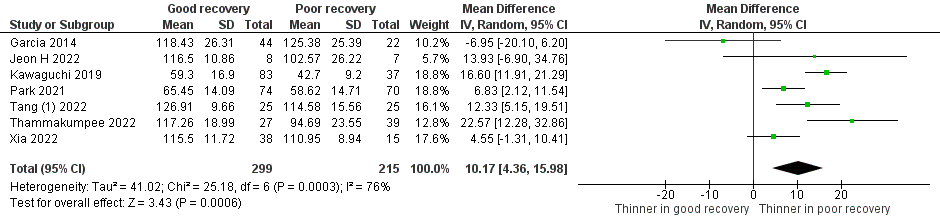


Supplementary figure 54 - MD - good outcome vs poor outcome – inferior pRNFL – Forest plot


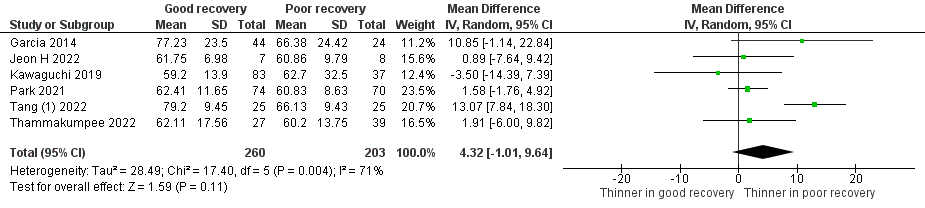


Supplementary figure 55 - MD - good outcome vs poor outcome – nasal pRNFL – Forest plot


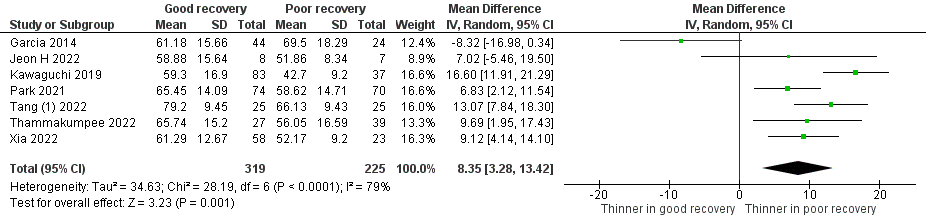


Supplementary figure 56 - MD - good outcome vs poor outcome – temporal pRNFL – Forest plot


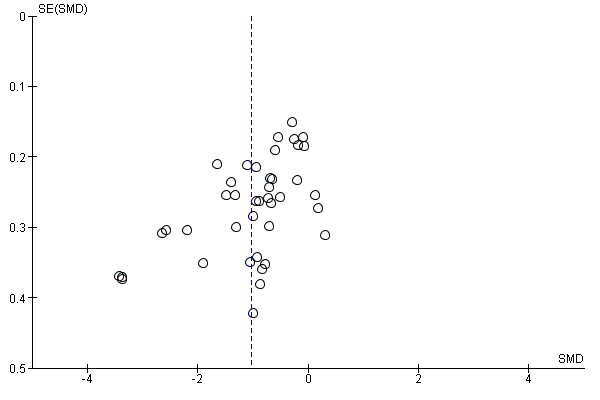


Supplementary figure 57 - SMD - healthy vs patients – mean pRNFL – Funnel plot


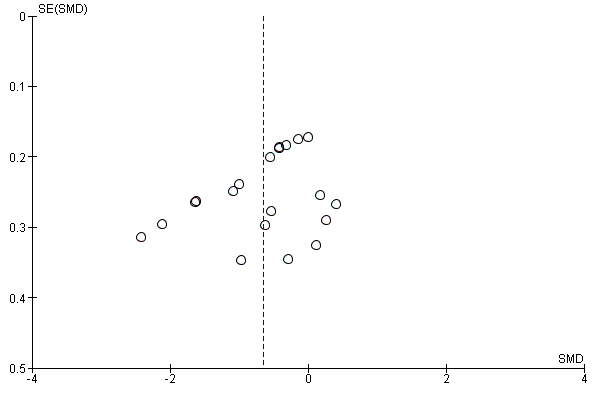


Supplementary figure 58 - SMD - healthy vs patients – superior pRNFL – Funnel plot


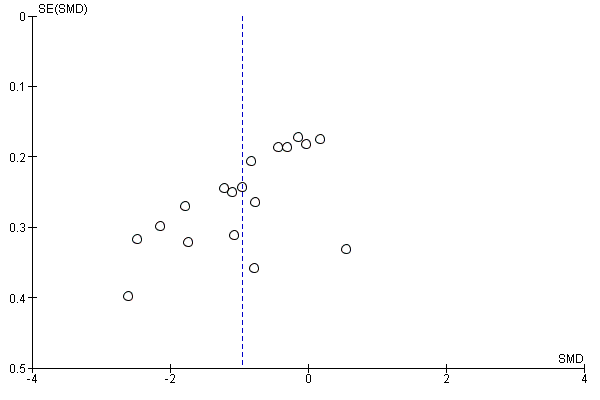


Supplementary figure 59 - SMD - healthy vs patients – inferior pRNFL – Funnel plot


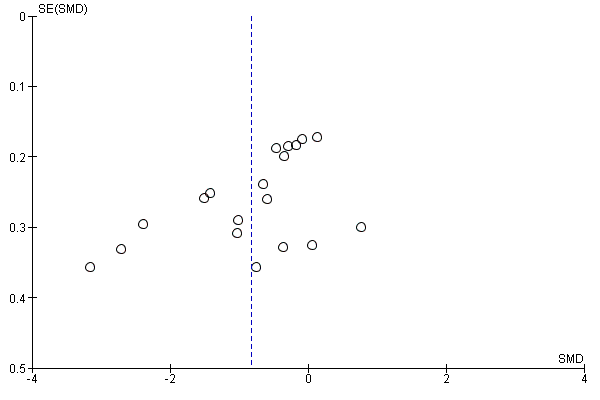


Supplementary figure 60 - SMD - healthy vs patients – nasal pRNFL – Funnel plot


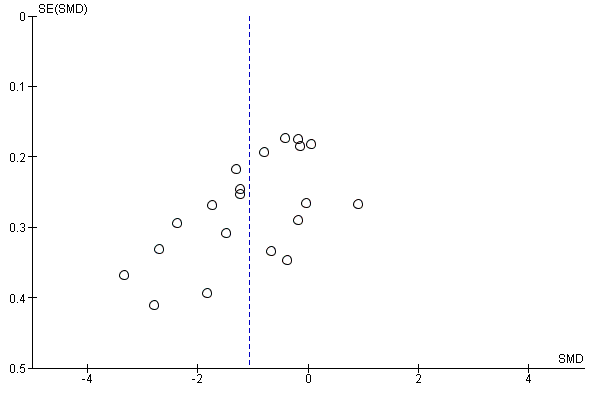


Supplementary figure 61 - SMD - healthy vs patients – temporal pRNFL – Funnel plot


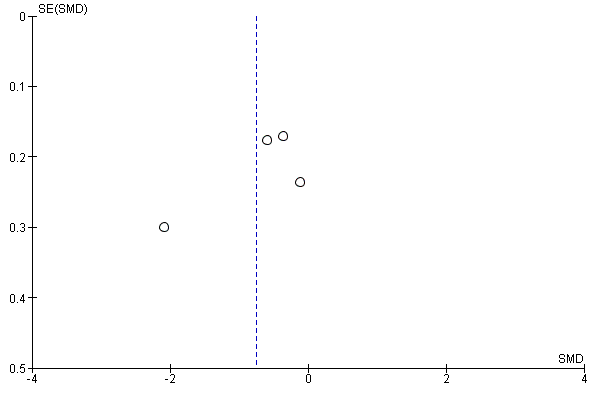


Supplementary figure 62 - SMD - healthy vs patients – nasal pRNFL (6 sector) – Funnel plot


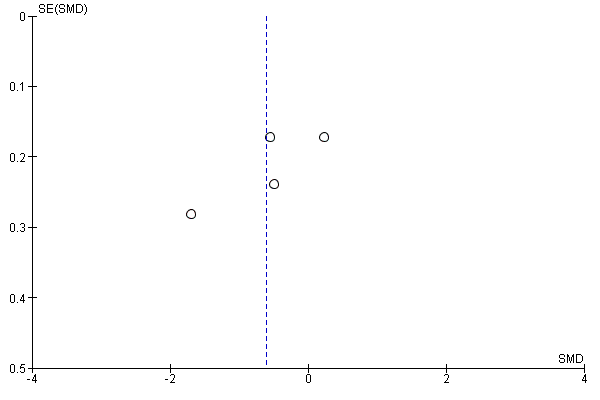


Supplementary figure 63 - SMD - healthy vs patients – naso-superior pRNFL (6 sector) – Funnel plot


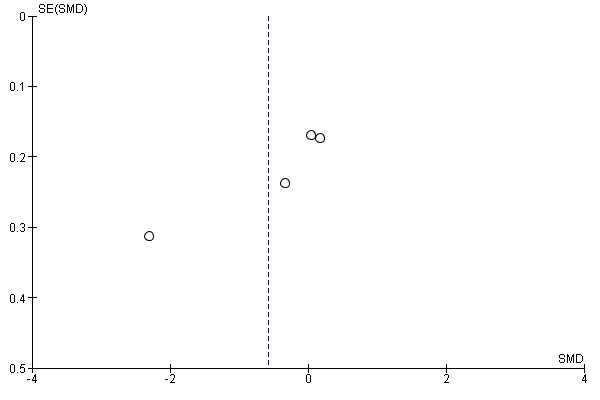


Supplementary figure 64 - SMD - healthy vs patients – naso-inferior pRNFL (6 sector) – Funnel plot


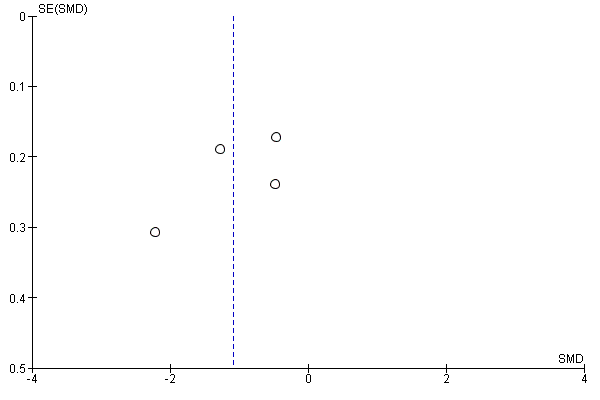


Supplementary figure 65 - SMD - healthy vs patients – temporal pRNFL (6 sector) – Funnel plot


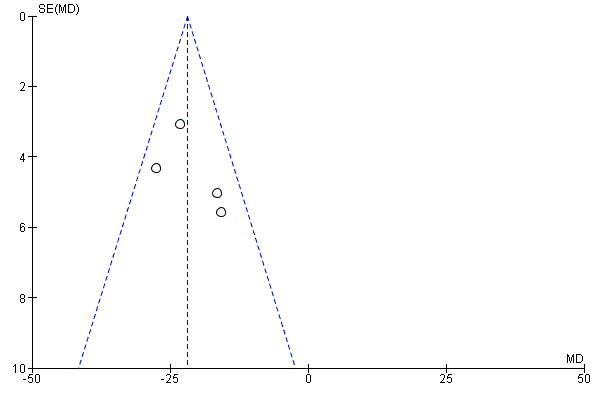


Supplementary figure 66 - SMD - healthy vs patients – temporo-superior pRNFL (6 sector) – Funnel plot


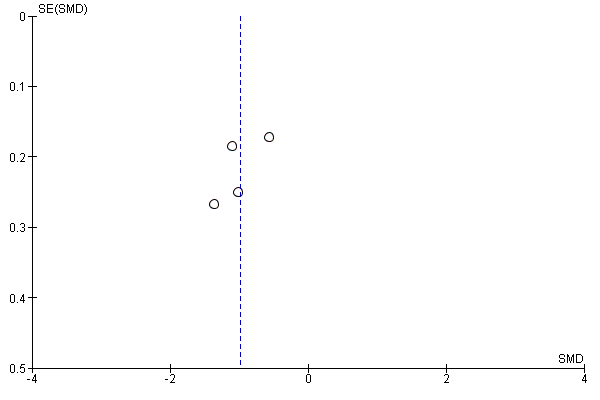


Supplementary figure 67 - SMD - healthy vs patients – temporo-inferior pRNFL (6 sector) – Funnel plot


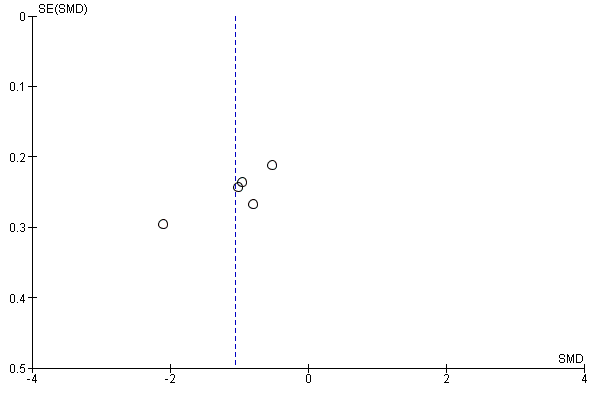


Supplementary figure 68 - SMD - healthy vs patients – mean mRNFL – Funnel plot


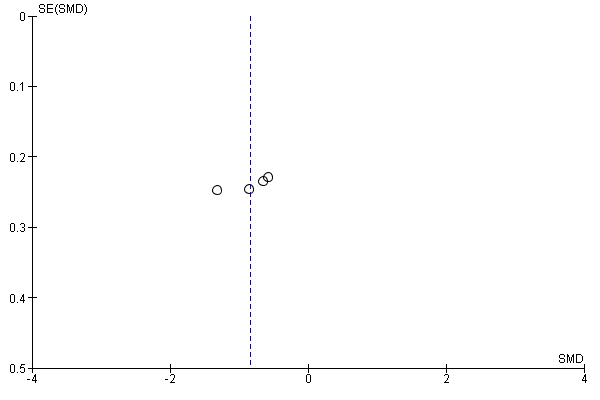


Supplementary figure 69 - SMD - healthy vs patients – supero-temporal mRNFL (box) – Funnel plot


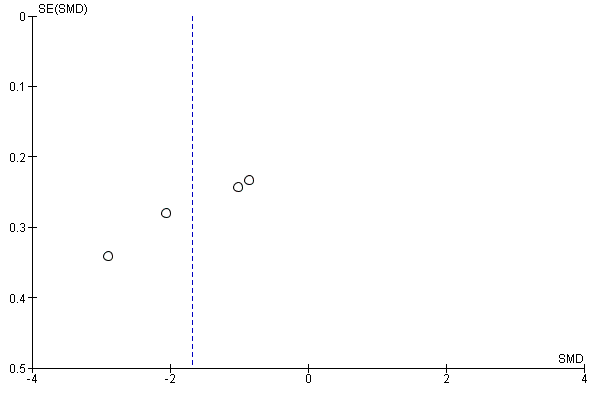


Supplementary figure 70 - SMD - healthy vs patients – supero-nasal mRNFL (box) – Funnel plot


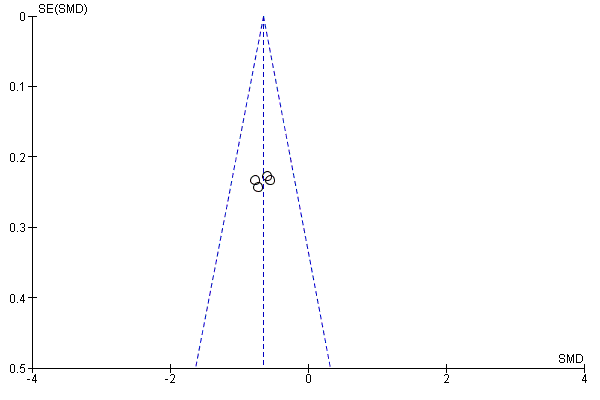


Supplementary figure 71 - SMD - healthy vs patients – infero-temporal mRNFL (box) – Funnel plot


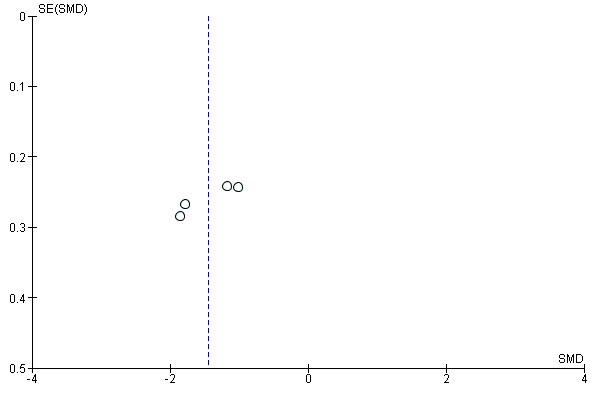


Supplementary figure 72 - SMD - healthy vs patients – infero-nasal mRNFL (box) – Funnel plot


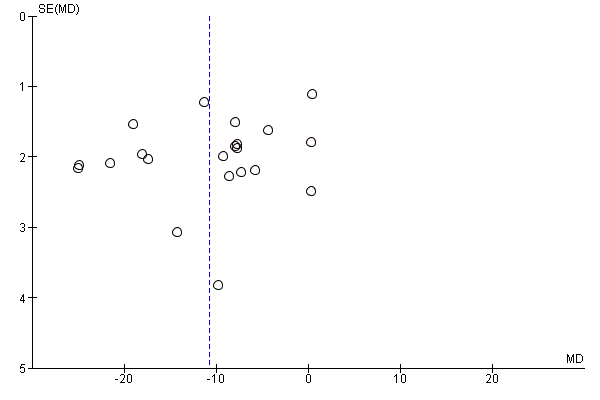


Supplementary figure 73 - SMD - healthy vs patients – mean mGCC – Funnel plot


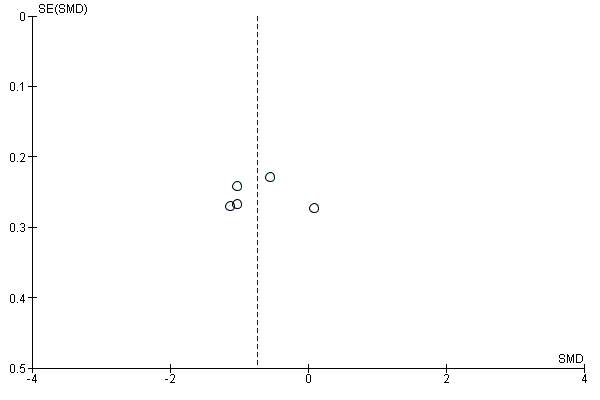


Supplementary figure 74 - SMD - healthy vs patients – superior mGCC – Funnel plot


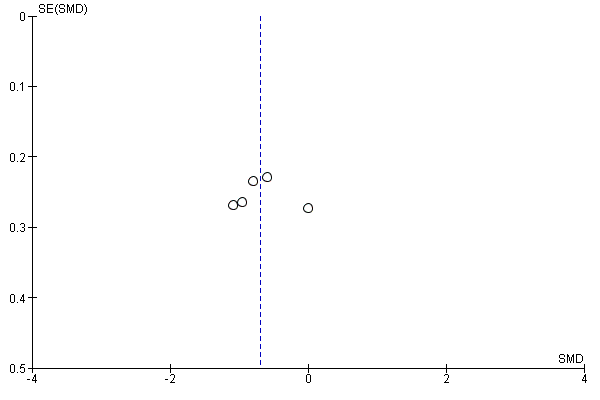


Supplementary figure 75 - SMD - healthy vs patients – inferior mGCC – Funnel plot


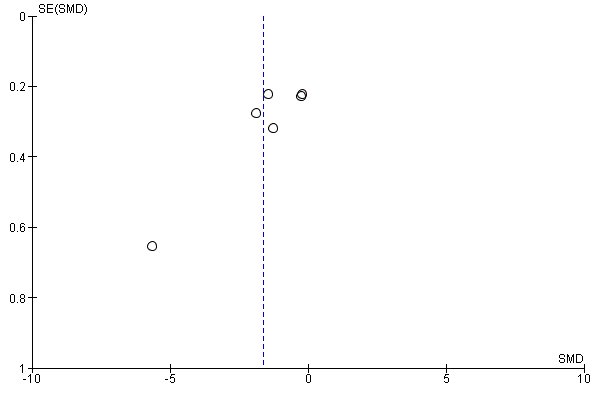


Supplementary figure 76 - SMD - healthy vs patients – mean mGCIPL – Funnel plot


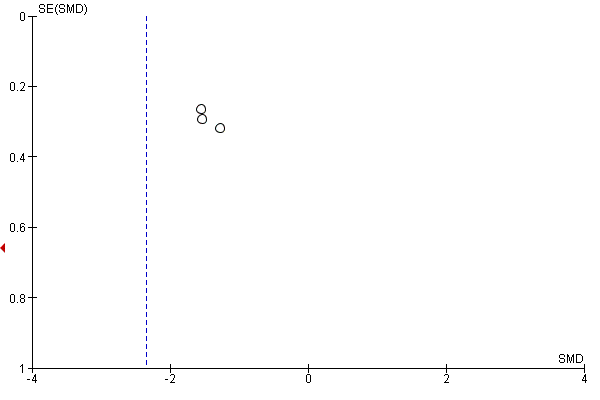


Supplementary figure 77 - SMD - healthy vs patients – superior mGCIPL (6 sector) – Funnel plot


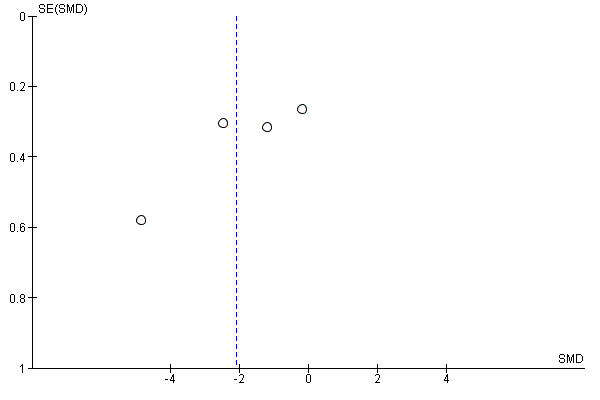


Supplementary figure 78 - SMD - healthy vs patients – supero-nasal mGCIPL (6 sector) – Funnel plot


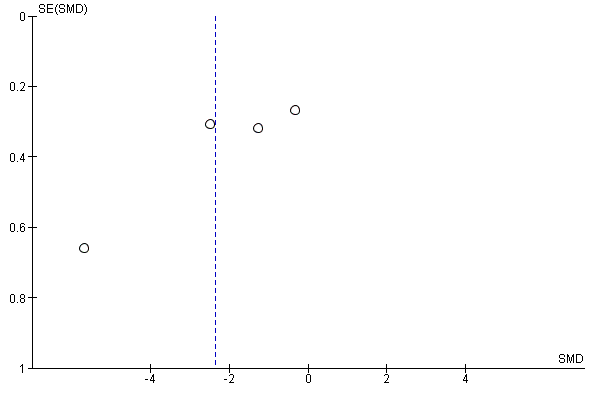


Supplementary figure 79 - SMD - healthy vs patients – infero-nasal mGCIPL (6 sector) – Funnel plot


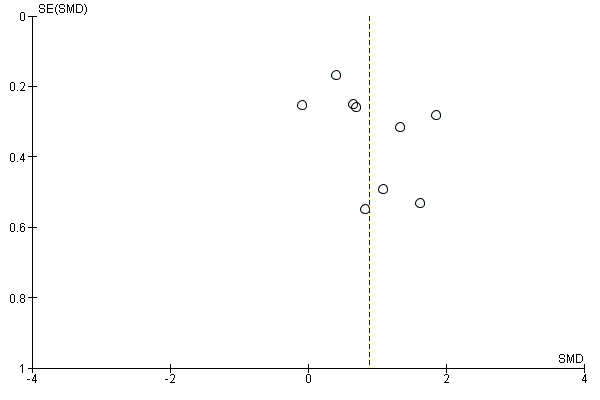


Supplementary figure 80 - SMD - good outcome vs poor outcome – mean pRNFL – Funnel plot


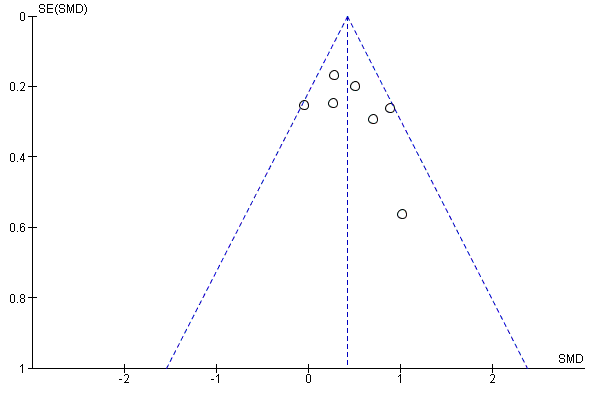


Supplementary figure 81 - SMD – good outcome vs poor outcome – superior pRNFL – Funnel plot


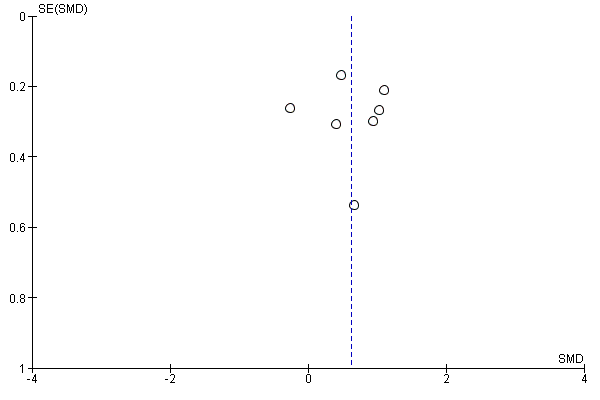


Supplementary figure 82 - SMD – good outcome vs poor outcome – inferior pRNFL – Funnel plot


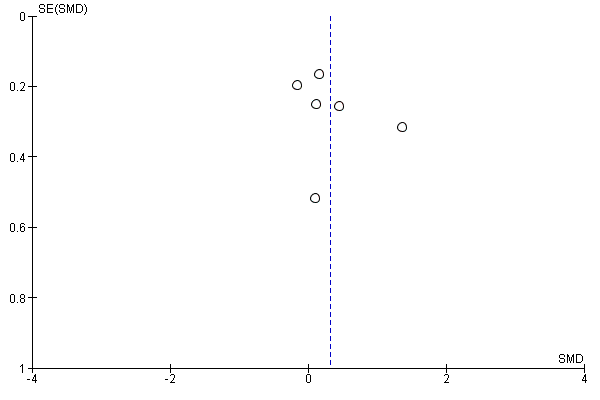


Supplementary figure 83 - SMD - good outcome vs poor outcome – nasal pRNFL – Funnel plot


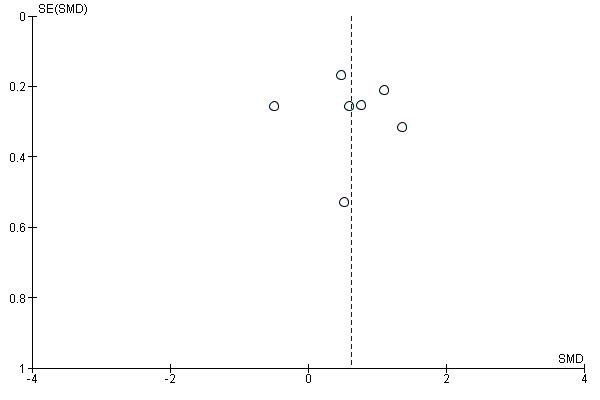


Supplementary figure 84 - SMD - good outcome vs poor outcome – temporal pRNFL – Funnel plot


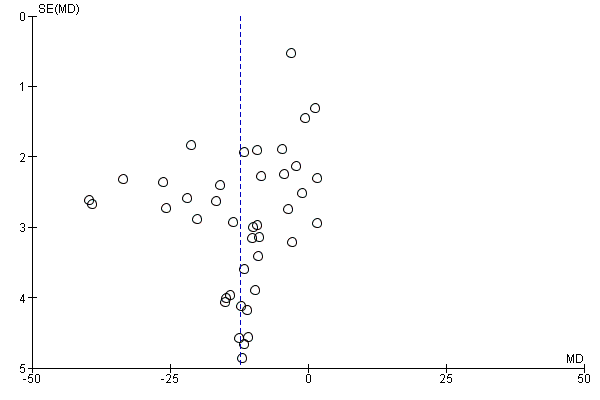


Supplementary figure 85 - MD - healthy vs patients – mean pRNFL – Funnel plot


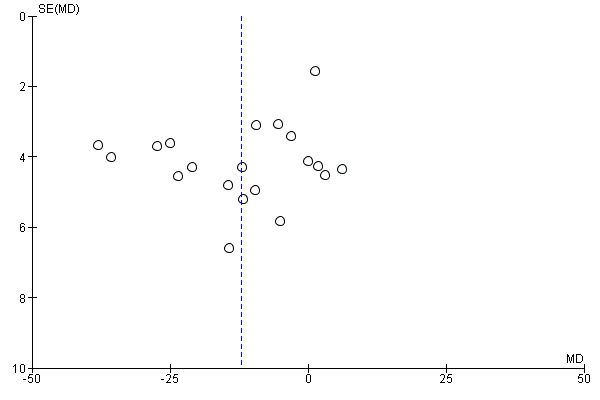


Supplementary figure 86 - MD - healthy vs patients – superior pRNFL – Funnel plot


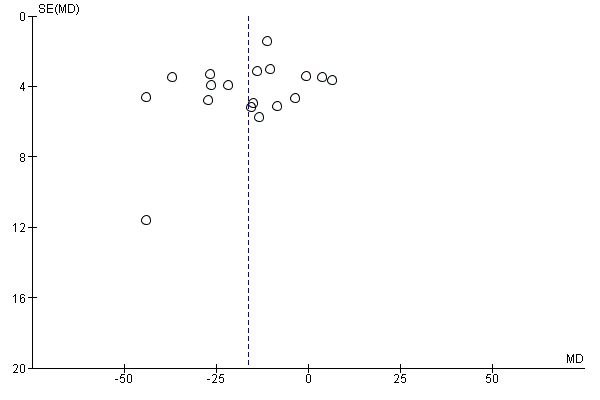


Supplementary figure 87 - MD - healthy vs patients – inferior pRNFL – Funnel plot


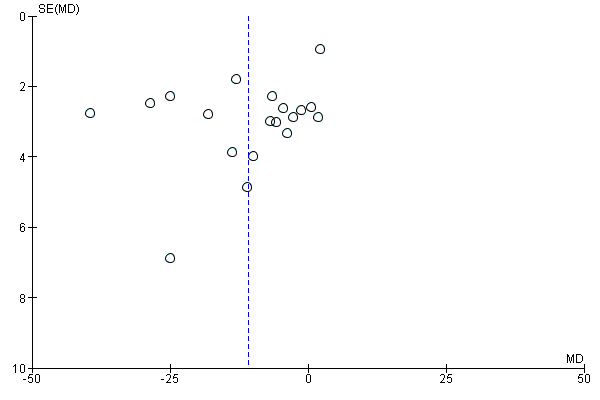


Supplementary figure 88 - MD - healthy vs patients – nasal pRNFL – Funnel plot


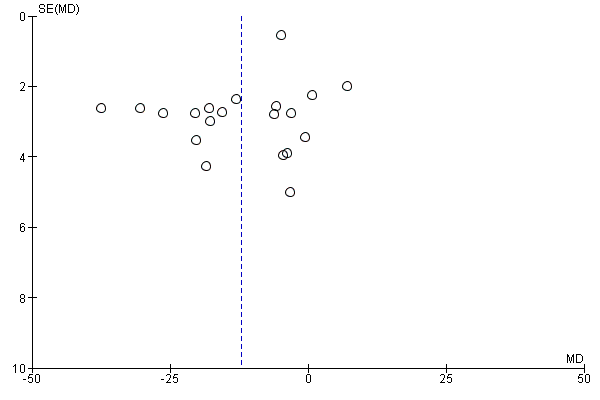


Supplementary figure 89 - MD - healthy vs patients – temporal pRNFL – Funnel plot


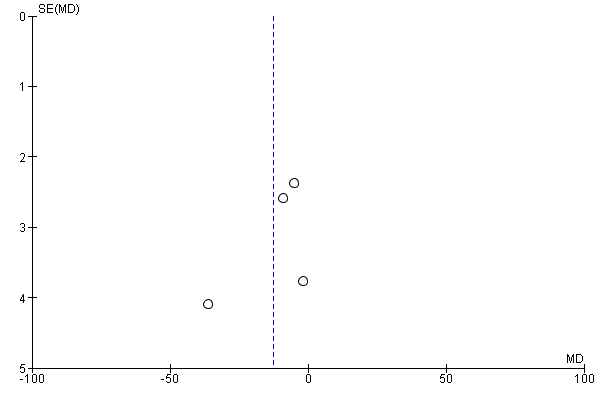


Supplementary figure 90 - MD - healthy vs patients – nasal pRNFL (6 sector) – Funnel plot


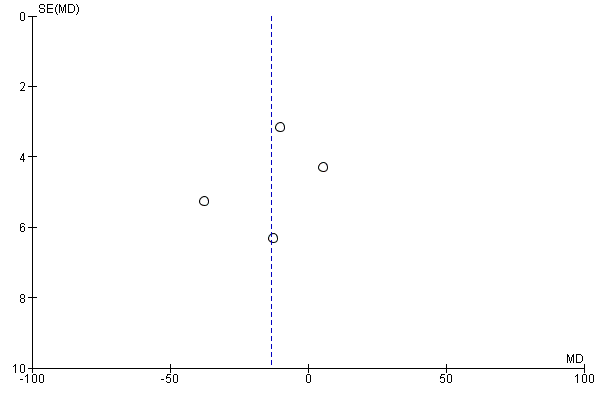


Supplementary figure 91 - MD - healthy vs patients – naso-superior pRNFL (6 sector) – Funnel plot


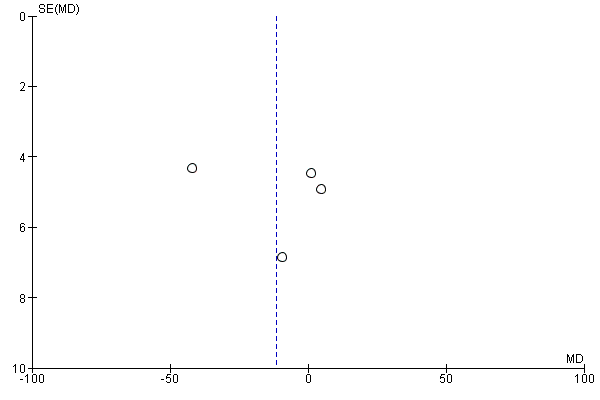


Supplementary figure 92 - MD - healthy vs patients – naso-inferior pRNFL (6 sector) – Funnel plot


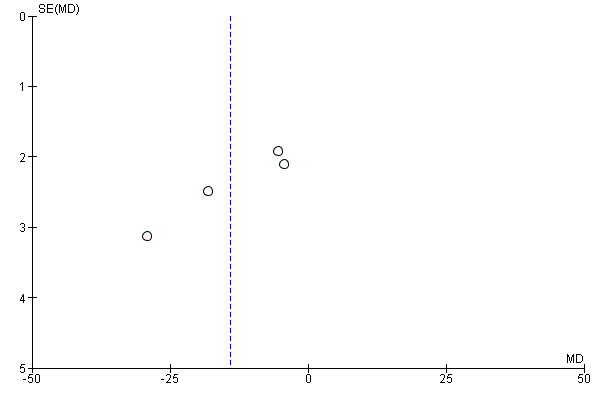


Supplementary figure 93 - MD - healthy vs patients – temporal pRNFL (6 sector) – Funnel plot


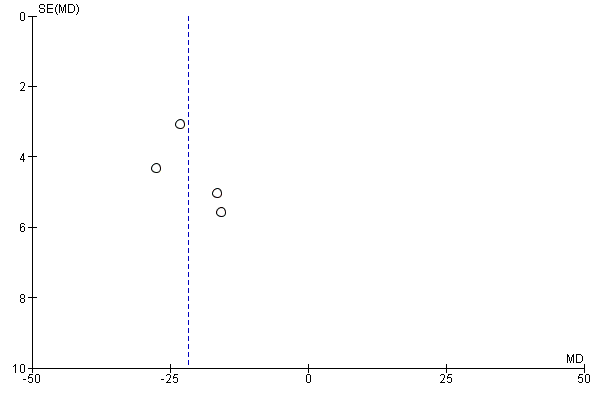


Supplementary figure 94 - MD - healthy vs patients – temporo-superior pRNFL (6 sector) – Funnel plot


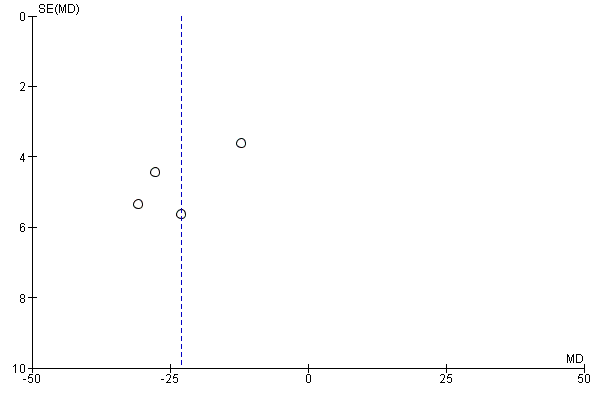


Supplementary figure 95 - MD - healthy vs patients – temporo-inferior pRNFL (6 sector) – Funnel plot


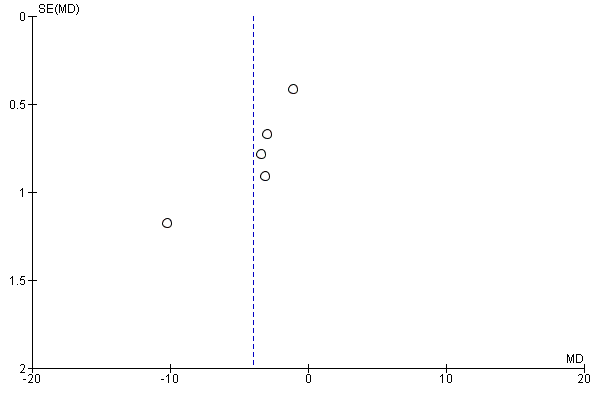


Supplementary figure 96 - MD - healthy vs patients – mean mRNFL – Funnel plot


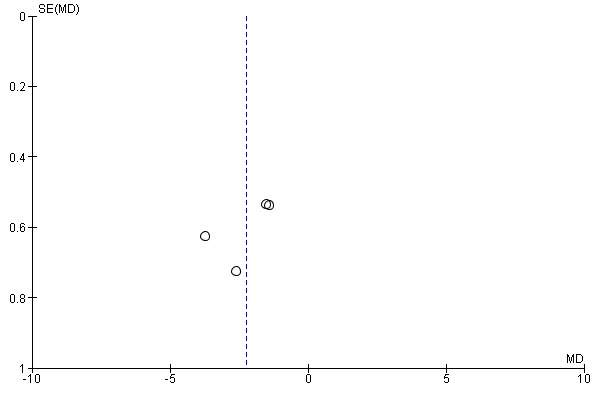


Supplementary figure 97 - MD - healthy vs patients – supero-temporal mRNFL (box) – Funnel plot


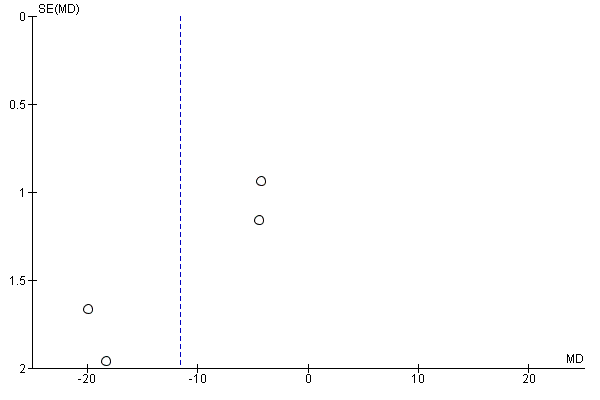


Supplementary figure 98 - MD - healthy vs patients – supero-nasal mRNFL (box) – Funnel plot


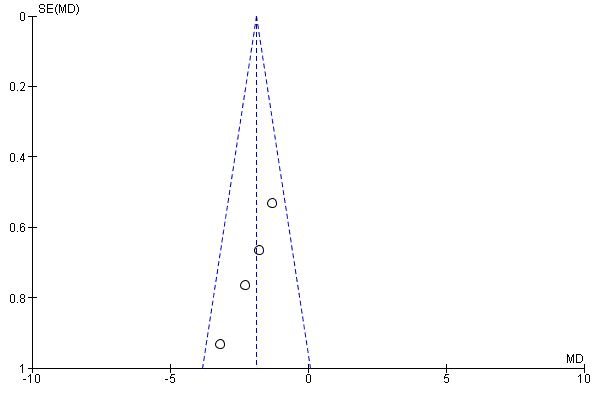


Supplementary figure 99 - MD - healthy vs patients – infero-temporal mRNFL (box) – Funnel plot


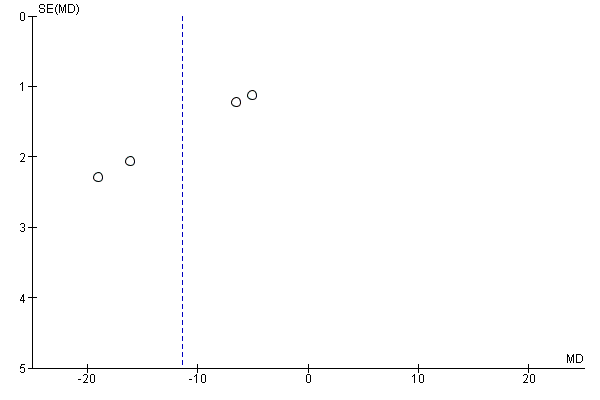


Supplementary figure 100 - MD - healthy vs patients – infero-nasal mRNFL (box) – Funnel plot

Supplementary figure 101 - MD - healthy vs patients – mean mGCC – Funnel plot

Supplementary figure 102 - MD - healthy vs patients – superior mGCC – Funnel plot

Supplementary figure 103 - MD - healthy vs patients – inferior mGCC – Funnel plot

Supplementary figure 104 - MD - healthy vs patients – mean mGCIPL – Funnel plot

Supplementary figure 105 - MD - healthy vs patients – superior mGCIPL (6 sector) – Funnel plot

Supplementary figure 106 - MD - healthy vs patients – supero-nasal mGCIPL (6 sector) – Funnel plot

Supplementary figure 107 - MD - healthy vs patients – infero-nasal mGCIPL (6 sector) – Funnel plot

Supplementary figure 108 - MD - good outcome vs poor outcome – mean pRNFL – Funnel plot

Supplementary figure 109 - MD - good outcome vs poor outcome – superior pRNFL – Funnel plot

Supplementary figure 110 – MD - good outcome vs poor outcome – inferior pRNFL – Funnel plot

Supplementary figure 111 - MD - good outcome vs poor outcome – nasal pRNFL – Funnel plot

Supplementary figure 112 - MD - good outcome vs poor outcome – temporal pRNFL – Funnel plot

Supplementary figure 113 – Methodological quality summary

Supplementary figure 114 – methodological quality graph
